# Tracking migraine outcomes over time by age and monoclonal antibody type: a retrospective, single-center, real-world study

**DOI:** 10.3389/fneur.2026.1893551

**Published:** 2026-08-11

**Authors:** Irene Mattioli, Alessandro Rondina, Alice Lanfranchi, Giulia Ceccardi, Michele Di Pasquale, Elena Cresta, Andrea Pilotto, Renata Rao, Alessandro Padovani

**Affiliations:** 1 Neurology Unit, Department of Frailty and Continuity of Care, ASST Spedali Civili, Brescia, Italy; 2 Neurology Unit, Department of Clinical and Experimental Sciences, University of Brescia, Brescia, Italy; 3 Neurology Unit, ASST Cremona, Cremona, Italy

**Keywords:** chronic migraine, disability, episodic migraine, headache, preventive treatment

## Abstract

**Background:**

Calcitonin gene-related peptide (CGRP) monoclonal antibodies (mAbs) are established preventive treatments for episodic and chronic migraine, but comparative longitudinal data across agents and age groups remain limited. This study aimed to evaluate long-term responses to different CGRP mAbs, assess whether age or treatment type influences response trajectories, and characterize multidimensional patterns of clinical response.

**Methods:**

We conducted a retrospective, single-center observational study including 453 adults with episodic or chronic migraine treated with erenumab, galcanezumab, fremanezumab, or eptinezumab between November 2018 and April 2026. Patients were stratified by age (<50 vs. ≥ 50 years) and different CGRP mAbs treatment. Longitudinal mixed-effects models assessed changes in monthly migraine days, acute medication use, pain intensity, disability, quality of life, and mood over 12 months. Generalized linear mixed models evaluated predictors of ≥50% response. Principal component analysis (PCA) was used to identify multidomain response patterns.

**Results:**

All CGRP mAbs produced rapid and sustained improvements across migraine frequency, disability, and patient-reported outcomes over 12 months. Age did not significantly influence baseline severity, longitudinal trajectories, or responder status. Treatment differences were modest overall; however, at 12 months, fremanezumab and eptinezumab were associated with higher odds of response compared with erenumab, while galcanezumab showed consistently higher response odds independent of time. PCA identified a *General improvement* component capturing multidomain benefit, which was more pronounced in the eptinezumab group.

**Conclusion:**

In real-world clinical practice, CGRP mAbs demonstrate comparable and durable effectiveness across age groups. Subtle long-term differences between agents may emerge, particularly for multidimensional clinical improvement, supporting individualized treatment selection.

## Background

Migraine is a major contributor to global population ill health (1), and migraine care has been greatly improved with the availability of monoclonal antibodies (mAbs) for prevention of episodic and chronic migraine (EM and CM) in adults (2).

The rationale for these agents is based on the central role of the calcitonin gene-related peptide (CGRP) in migraine pathophysiology: CGRP is a neuropeptide involved in trigeminovascular pathway activation, vasodilation, and pain transmission during migraine attacks (3, 4). The mAbs either target the CGRP ligand (fremanezumab, galcanezumab, eptinezumab) or the CGRP receptor (erenumab), thereby inhibiting the CGRP pathway.

Pooled analyses and head-to-head studies consistently show similar reductions in monthly migraine days (MMD) and disability scores across these agents, with no significant superiority of one over another in either EM or CM populations (5–9). In over 5,800 migraine patients treated with anti-CGRP mAbs, it has been shown that lower baseline disability, less MMD, and absence of depression were associated with higher odds of achieving good or excellent responses (10). These findings highlight that anti-CGRP mAbs not only reduce migraine frequency, but also ameliorate disabling associated symptoms, improve quality of life, and that earlier initiation before severe chronification may maximize benefits.

The growing emphasis on early initiation of CGRP mAbs, together with their favorable safety and tolerability profile, supports their widespread and prolonged use. This trend raises important considerations regarding the aging of treated populations and the broader general demographic shift toward older patients, for whom clinical trial data remain limited. The pivotal randomized controlled trials for erenumab, galcanezumab, and fremanezumab excluded adults older than 65 at screening (11–14). Eptinezumab phase 3 trials included patients up to age 65, but *post hoc* analyses specifically evaluated efficacy (significant reduction in migraine frequency was achieved by 57.9% of eptinezumab-treated versus 35.7% of patients who received placebo) and safety in patients aged ≥50 years, with the oldest included being 71 years old (15). Real-world studies and postmarketing analyses have included older adults, reporting use in patients up to age 87 for erenumab, galcanezumab, and fremanezumab, showing comparable safety (constipation was more frequent for erenumab) and efficacy (the proportion of patients obtaining >50% reduction in migraine frequency was 57%) to younger populations (16–18). For eptinezumab, postmarketing safety data include adults up to 64 years, and the most frequently reported adverse drug reactions were fatigue and injection site reactions (19).

Expanding our previous work on the efficacy of different CGRP agents (20), the present study was designed to assess longitudinal differences in clinical responses to CGRP mAbs in a real-world cohort, with a focus on head-to-head comparisons between treatment types—rather than pooling them together, and including the most recently available eptinezumab— and on the influence of age. Specifically, we aimed to: (i) evaluate whether clinical responses to CGRP mAbs vary over time according to patient age or treatment type; (ii) determine whether treatment type or age group predicts differential response trajectories over time; and (iii) characterize distinct patterns of clinical response to CGRP mAbs across different age groups.

## Methods

### Study design and participants

The present work is a retrospective observational study. The study population was selected from the clinical records of the Headache Centre of the Neurology Unit at “Spedali Civili” Hospital in Brescia (Italy). Consecutive patients who initiated treatment with an anti-CGRP monoclonal antibody at our center between November 2018 and April 2026 were included in the study and subsequently followed up according to routine clinical practice.

This study included all adult patients (age ≥18) with a diagnosis of EM (codes 1.1 and 1.2) or CM (code 1.3) according to the International Classification of Headache Disorders III (ICHD-III) (21) in prophylactic treatment with anti-CGRP mAbs (erenumab 140 mg monthly, galcanezumab 240 mg loading dose followed by 120 mg monthly, fremanezumab 225 mg monthly or 675 mg quarterly, and eptinezumab 100 mg, with the option to increase to 300 mg if response was insufficient after 2 infusions).

Erenumab was chosen as the reference comparator in all analyses, as it was the first CGRP mAb approved and has been evaluated in head-to-head trials (22, 23).

Inclusion criteria were the following: documented history of migraine for at least 12 months; patient’s daily paper headache diary compilation in the 3 months prior to study enrolment; ≥8 migraine days with Migraine Disability Assessment (MIDAS) ≥ 11 per month for at least 3 months; eligible participants had previously received other migraine prophylactic treatments and had shown an insufficient response after at least 6 weeks of therapy, or had demonstrated intolerance or clear contraindications to at least three different classes of migraine prophylactic medications (antidepressants, antiepileptics, beta-blockers, onabotulinumtoxinA). This assessment was based on the patients’ entire available lifetime treatment history prior to initiation of anti-CGRP monoclonal antibody therapy. These eligibility requirements corresponded to those defined by the Italian Medicines Agency (AIFA) for the prescription and reimbursement of these mAbs, and all the patients included in the present study met them. Because the number of patients with prior different anti-CGRP treatment exposure was limited, separating them into an additional subgroup would have reduced statistical power and increased the risk of unstable estimates. Therefore, these patients were kept in the main cohort.

Clinical and demographic information were collected for all patients at baseline (T0) and included: sex, education (years), ICHD-3 diagnosis (EM or CM), disease duration, number of previous prophylaxis treatments. To ensure comparable group sizes and consistency with prior evidence, including a *post hoc* analysis of eptinezumab (19), patients were stratified into two age groups: Under 50 (<50 years old) and Over 50 (≥50 years old).

Migraine-related variables were collected at T0 and following three, six and twelve months of treatment (T3, T6, T12) and included: headache frequency expressed as MMD, analgesics intake (monthly number of pills used, usually triptans, NSAIDs, combined analgesics, e.g., paracetamol+caffeine), attack pain intensity [using the Numerical Rating Scale, NRS (24)], measures of migraine disability [MIDAS (25), Headache Impact Test-6, HIT-6 (26)], quality of life [Short Form 36, SF36 (27)], depression [Beck Depression Inventory, BDI (28)], and anxiety [Zung’s Self-rating Anxiety Scale (29)]. MMD and monthly number of analgesics used were recorded by patients in headache diaries, either in electronic or paper format. All other clinical measures were patient-reported outcomes: patients completed the scales on paper the day before each visit. During the first visit, patients received instructions on how to complete the scales and could review or revise them with clinicians at each subsequent visit if they had any doubts. In this study, SF-36 item responses were summed to create a raw aggregate health-related quality-of-life score, with higher scores indicating better health status; this questionnaire assesses eight domains: physical functioning, role physical, bodily pain, general health, vitality, social functioning, role emotional, and mental health, and evaluates the impact of health status on physical, psychological, and social well-being. This score was not transformed to the conventional 0–100 SF-36 domain scale to preserve the granularity of the original responses and minimize additional data manipulation, consistent with the data-driven nature of the subsequent analyses.

### Statistical analysis

Statistical significance was set at *p* < 0.05. Data analyses were carried out with R Statistical Software (30).

Continuous variables were described as mean and standard deviation (SD), and categorical variables were expressed as percentages. Continuous variables were compared across age groups in different treatment groups using Student’s *t*-test or non-parametric tests (Kruskal–Wallis). Homogeneity of variances was assessed using Levene’s test. Group differences in categorical variables were assessed using Fisher’s exact test.

#### Clinical response across different age and treatment groups

We assessed whether clinical outcomes changed over follow-up as a function of age or treatment using longitudinal mixed-effects models on repeated measurements of MMD, acute medication use, NRS pain intensity, MIDAS, SF36, HIT-6, BDI, and Zung scores collected at T0, T3, T6, and T12. Where needed to improve distributional assumptions, outcomes were log-transformed.

For each endpoint, we fitted a separate linear mixed-effects model including fixed effects for Time, Age, Treatment, and their interactions, and a subject-specific random intercept to account for within-patient correlation. For outcomes showing serial dependence, temporal autocorrelation was modeled with a first-order autoregressive structure [AR(1)] defined on months within subject. When indicated by residual diagnostics, heteroscedasticity was accommodated through variance-function structures.

To improve robustness of inference, standard errors and tests for fixed effects were additionally obtained using cluster-robust (CR2) variance–covariance estimators at the patient level. Model adequacy was evaluated through diagnostics of collinearity, heteroscedasticity, residual normality, and residual autocorrelation.

As a sensitivity analysis, the same models were also run restricted to participants with complete follow-up data.

#### Prediction of treatment response

To evaluate whether age or treatment group predicted differences in response trajectories over time, a generalized linear mixed-effects model (GLMM) with a logit link was fitted. The dependent variable was responder status at each follow-up visit, defined as ≥ 50% reduction in MMD. Fixed effects included Treatment (reference = erenumab), Time (reference = T3), and Age (reference = Under 50), together with all Treatment × Time interaction terms. Because dose modifications occurred only for the galcanezumab (loading dose) and eptinezumab (possibility to increase dose if insufficient clinical response) groups, a time-varying covariate for dose was included to adjust for within-treatment dose variation. Dose was coded numerically for treatments with variable dosing and set to zero for fixed-dose treatments; the resulting variable was then cantered around the overall mean to improve numerical stability. A random intercept for each subject accounted for within-subject correlation across repeated measures. Model results are reported as odds ratios (OR) with 95% confidence intervals (CI). As a sensitivity analysis, the responder model was repeated with age included as a continuous variable rather than a categorical variable (<50 vs. ≥50 years) to assess the robustness of the findings to the specification of age. Another sensitivity analysis was run to assess the potential impact of confounding by indication: the longitudinal model was rerun with additional adjustment for baseline migraine severity (MMD at baseline), migraine diagnosis (EM or CM), number of previous preventive treatment failures, and disease duration (years).

An exploratory sub-analysis focused on older participants aged ≥65 years (*n* = 15). Due to the limited subgroup size, a simplified GLMM with random intercepts for participants was fitted, including Time (T0, T6, T12), age ≥65, and their interaction, without adjustment for treatment group.

#### Patterns of clinical outcomes

To characterize distinct patterns of clinical response to CGRP mAbs across disability, mood, and quality-of-life domains, we analyzed longitudinal change scores. For each participant, change values (*Δ*) at 3, 6, and 12 months were calculated relative to baseline for all clinical measures (MIDAS, HIT-6, Zung, BDI, SF36). All Δ scores were standardized (z-score), and a principal component analysis (PCA) was used to summarize shared variance across correlated change measures and identify latent patterns of multidomain response. The number of components to retain was determined using multiple criteria: eigenvalues (Kaiser criterion ≥1), inspection of the scree plot, cumulative explained variance (threshold of 40%). An oblimin rotation was applied to allow for correlation between components.

Because PCA scores summarize standardized within-person change across domains, they served as composite indicators of clinical response to CGRP mAbs. Group differences in component scores were examined using Kruskal–Wallis tests for age and treatment groups. When significant omnibus effects were detected, Dunn’s post-hoc tests with Bonferroni correction were performed to identify pairwise differences between treatment groups.

#### Missing data handling

Missing outcome data occurred not only because of treatment discontinuation, but mainly because of unavailable questionnaire data or because some patients, particularly in the eptinezumab group, had not yet completed 12 months of follow-up at the time of data extraction. Supplementary Table 1 reports the number of available observations for each outcome at each timepoint. To minimize loss of information, all available observations were retained in the longitudinal analyses. Mixed-effects models allow inclusion of participants with partially observed follow-up data and use all available repeated measurements to contribute to model estimation, maximizing the statistical power while accounting for within-subject correlation over time. Sensitivity analyses restricted to participants with complete follow-up data were also performed. For the PCA of longitudinal change scores, missing values were handled using missMDA principal component imputation.

## Results

**Figure 1:**
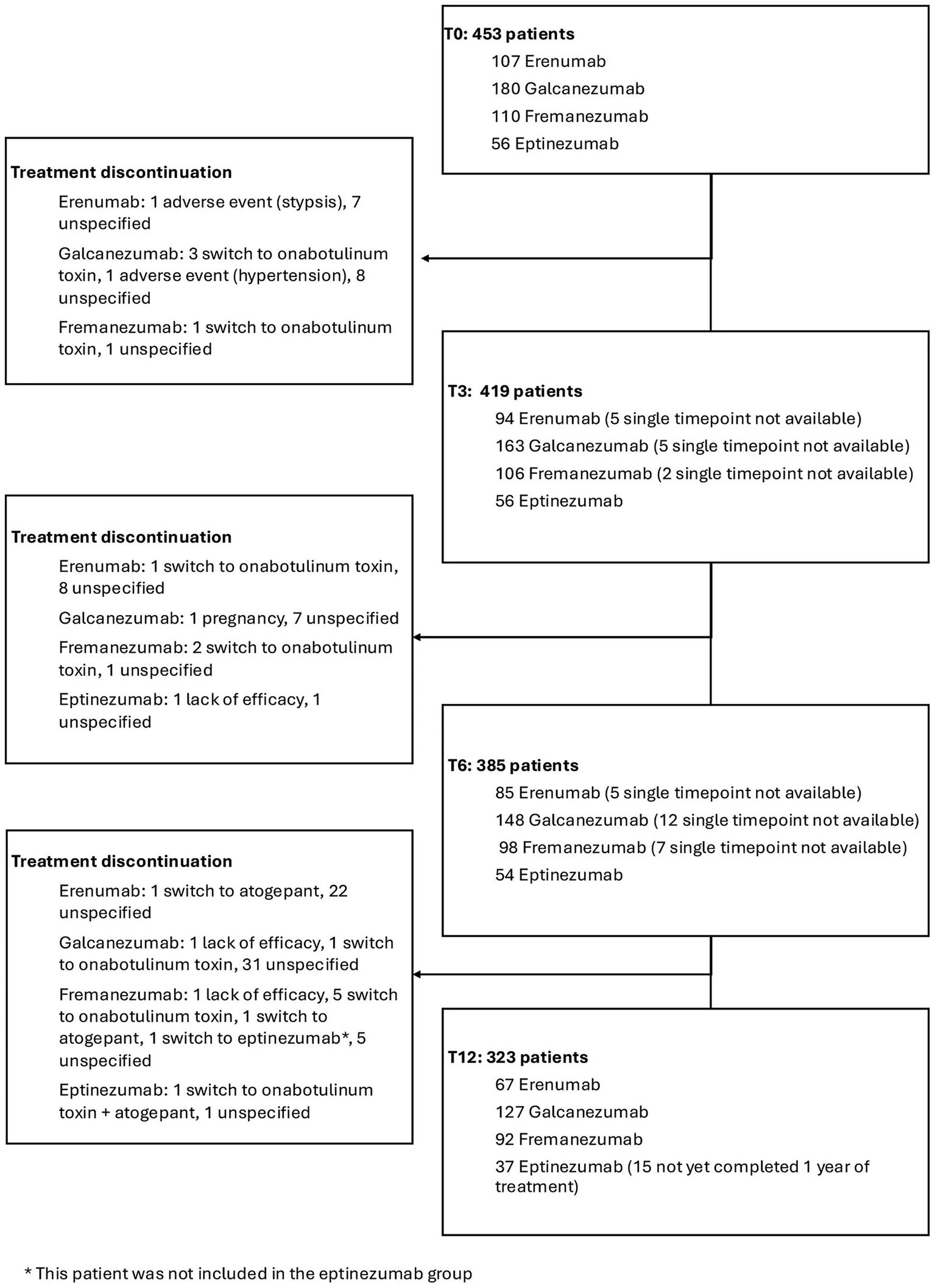
Overview flowchart of the follow-up over the 12-month. This figure was included to provide a transparent overview of patient inclusion, treatment discontinuation, and follow-up completion over time. To see how missing data were handled analytically, please refer to the dedicated part of the “Methods” section.

A total of 453 patients were included in the study, of whom 71% completed the 12-month follow-up. Figure 1 summarizes data availability for each timepoint and reasons for treatment discontinuation. Dropout rates for treatment discontinuation were 37% for erenumab, 29% for galcanezumab, 16% for fremanezumab, and 7% for eptinezumab. Table 1 reports the baseline demographic, clinical, and migraine-related characteristics of the enrolled participants. Patients’ prior different anti-CGRP treatment history is reported descriptively in Supplementary Table 2.

**Table 1:** Baseline demographic, clinical, and migraine-related characteristics.

Treatment group	Variable	Under 50	Over 50	*p*_value
Erenumab (*n* = 107)	Participants (*n*)	60	47	
	Age (years)	39 ± 8	56 ± 4	**<0.001**
	Sex	F 46 (77%) M 14 (23%)	F 41(87%) M 6 (13%)	0.2^§^
	Education (years)	14.0 ± 3.8	13.4 ± 3.9	0.5
	Diagnosis (CM)	39 (65%)	32 (68%)	0.8^§^
	Years with headache	21 ± 10	34 ± 12	**<0.001**
	Number of previous prophylaxis treatments	4.83 ± 2.44	4.87 ± 1.78	>0.9
	MMD	19 ± 8	18 ± 8	0.7
	NRS	7.93 ± 1.10	7.86 ± 1.09	0.8
	Monthly acute medications	20 ± 11	16 ± 10	0.08
	MIDAS	95 ± 76	81 ± 45	0.2
	SF36	105 ± 13	105 ± 9	0.9
	BDI	15 ± 12	13 ± 8	0.5
	Zung	38 ± 6	35 ± 7	**0.03**
	HIT-6	66 ± 8	66 ± 8	0.9
Galcanezumab (*n* = 180)	Participants (*n*)	126	54	
	Age	38 ± 8	57 ± 5	**<0.001**
	Sex	F110 (87%) M16 (13%)	F49 (91%) M 5(9%)	0.6^§^
	Education (years)	14.3 ± 3.3	13.0 ± 4.0	0.05
	Diagnosis (CM)	66 (52%)	30 (56%)	0.7^§^
	Years with headache	22 ± 9	36 ± 14	**<0.001**
	Number of previous prophylaxis treatments	3.02 ± 1.34	3.52 ± 1.26	**0.02**
	MMD	16 ± 7	17 ± 8	0.5
	NRS	7.73 ± 0.87	7.60 ± 1.21	0.5
	Monthly acute medications	19 ± 17	19 ± 20	0.8
	MIDAS	87 ± 59	90 ± 66	0.7
	SF36	103 ± 18	97 ± 33	0.2
	BDI	14 ± 10	11 ± 9	0.08
	Zung	37 ± 7	36 ± 7	0.3
	HIT-6	65 ± 6	64 ± 8	0.5
Fremanezumab (*n* = 110)	Participants (*n*)	60	50	
	Age	40 ± 7	59 ± 5	**<0.001**
	Sex	F 49 (82%) M 11 (18%)	F 37 (74%) M 13 (26%)	0.4^§^
	Education (years)	13.2 ± 3.0	11.7 ± 2.8	**0.007**
	Diagnosis (CM)	39 (65%)	40 (80%)	0.09^§^
	Years with headache	24 ± 9	36 ± 12	**<0.001**
	Number of previous prophylaxis treatments	3.15 ± 1.23	3.62 ± 1.46	0.07
	MMD	18 ± 8	18 ± 7	0.8
	NRS	7.60 ± 0.87	7.86 ± 0.89	0.1
	Monthly acute medications	17 ± 12	17 ± 13	0.9
	MIDAS	81 ± 50	92 ± 61	0.3
	SF36	105 ± 16	102 ± 23	0.5
	BDI	16 ± 10	12 ± 9	0.07
	Zung	40 ± 7	36 ± 7	**0.008**
	HIT-6	64.9 ± 5.8	65.3 ± 6.3	0.8
Eptinezumab (*n* = 56)	Participants (*n*)	36	20	
	Age	38 ± 9	56 ± 5	**<0.001**
	Sex	F 32 (89%) M 3 (11%)	F 18 (90%) M 3 (10%)	0.9^§^
	Education (years)	13.2 ± 2.2	13.7 ± 4.1	0.7
	Diagnosis (CM)	22 (61%)	10 (50%)	0.6^§^
	Years with headache	24 ± 10	38 ± 11	**<0.001**
	Number of previous prophylaxis treatments	3.67 ± 1.87	4.40 ± 2.04	0.2
	MMD	22 ± 8	23 ± 8	0.6
	NRS	7.83 ± 0.81	7.65 ± 1.14	0.5
	Monthly acute medications	22 ± 15	34 ± 20	**0.03**
	MIDAS	89 ± 55	100 ± 67	0.5
	SF36	105.1 ± 6.5	107.5 ± 6.2	0.2
	BDI	17 ± 11	15 ± 12	0.6
	Zung	39 ± 8	37 ± 8	0.4
	HIT-6	64 ± 7	61 ± 11	0.2

Results for continuous variables are expressed as mean±standard deviation (SD). Categorical variables are expressed as numerosity (percentage of the group total). *p* values refer to Student’s *t* test across age groups, except ^§^ which refer to Fisher’s exact test. Significant *p* values are highlighted in bold.

BDI, Beck Depression Inventory; CM, Chronic Migraine; HIT-6, Headache Impact Test-6; MIDAS, Migraine Disability Assessment; MMD, Monthly Migraine Days; NRS, Numerical Rating Scale; SF36, Short Form 36.

### Clinical response across different treatment and age groups

**Figure 2:**
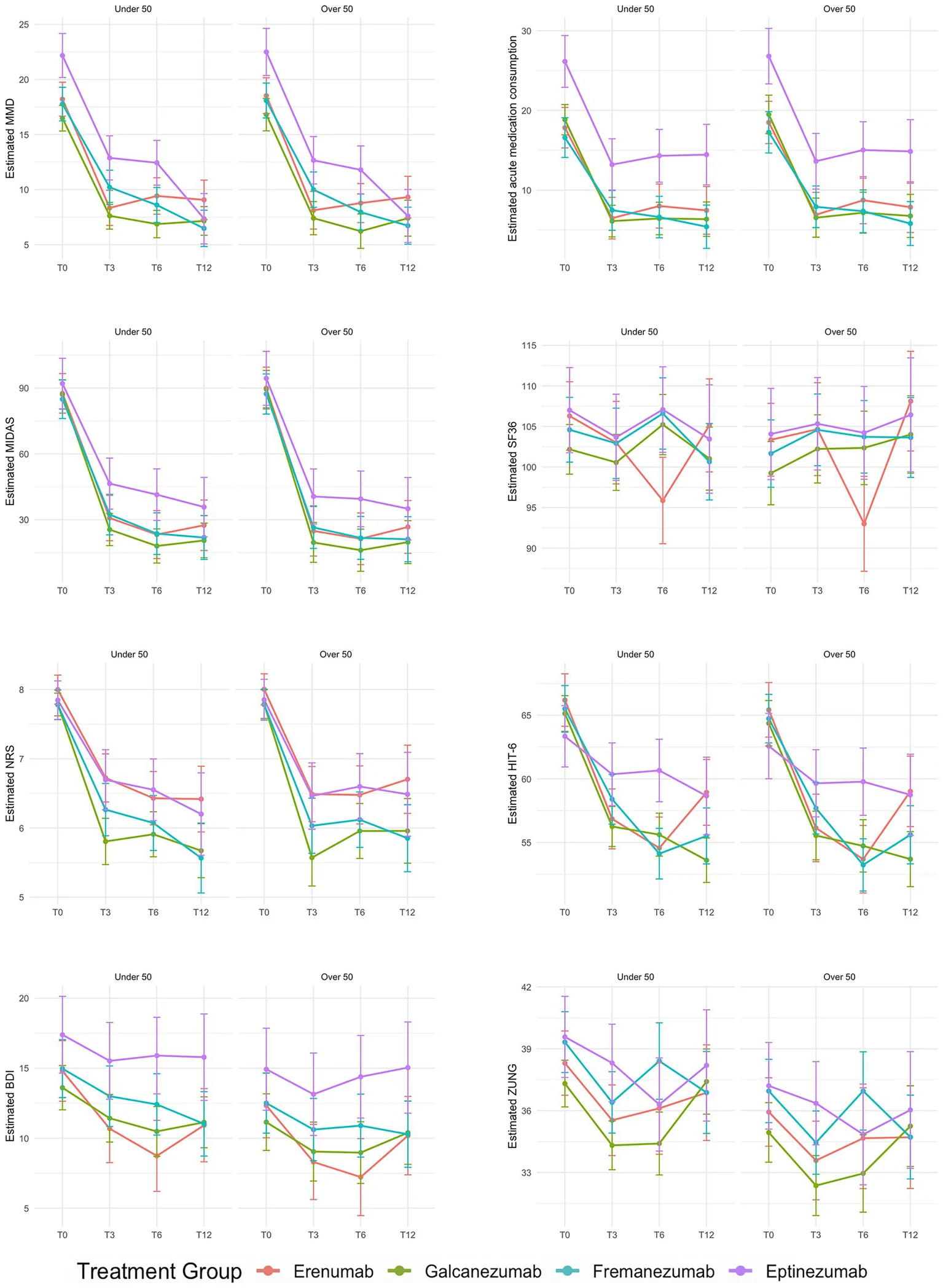
Clinical and migraine-related variables change over time across age and treatment groups. BDI, Beck Depression Inventory; HIT-6, Headache Impact Test-6; MIDAS, Migraine Disability Assessment; MMD, Monthly Migraine Days; NRS, Numerical Rating Scale; SF36, Short Form 36.

Across all mixed-effects models, treatment with CGRP mAbs was associated with rapid, substantial, and generally sustained clinical improvement over the 12-month follow-up period (Figure 2; Supplementary Table 3).

Patients experienced a marked reduction in migraine frequency, with decreases of −9.87 (95% CI −11.39 to −8.35) migraine days at 3 months, −8.79 (95% CI −10.37 to −7.21) migraine days at 6 months, and −9.14 (95% CI −10.86 to −7.41) migraine days at 12 months relative to baseline (all *p* < 0.001). This pattern indicates a strong early response that remained stable over time. Age neither affected baseline MMD nor modified treatment response. At baseline, eptinezumab-treated patients had higher MMD (3.96, *p* < 0.001). Longitudinally, fremanezumab demonstrated a slightly slower improvement at 3 months (2.32 migraine days, *p* = 0.02), with a nearly significant further improvement at T12 (−2.15, *p* = 0.05). Eptinezumab showed a trend toward greater 12-month improvement (−5.68 days, *p* < 0.001).

Acute medication consumption decreased robustly over time (−11.35, 95% CI −14.11 to −8.59, pills/month at 3 months, −9.83, 95% CI −12.74 to −6.92, pills/month at 6 months, −9.83, 95% CI −12.74 to −6.92, pills/month at 12 months; all *p* < 0.001). Age had no influence on baseline use or longitudinal response. Eptinezumab users presented with higher baseline acute medication intake (8.32 pills/month, *p* < 0.001), but no treatment exhibited differential change over time, suggesting convergent reductions in medication overuse across all groups.

Pain intensity showed a clinically meaningful decline across all timepoints (−1.28 to −1.58 in the total NRS score; all *p* < 0.001), with no age differences in baseline scores or treatment response. Although baseline NRS did not differ significantly by treatment, early differences emerged: galcanezumab showed greater improvement at 3 months (−0–70, *p* = 0.002), and fremanezumab demonstrated an additional reduction at 12 months (−0.063, *p* = 0.05). However, these treatment effects were not consistent across timepoints and did not suggest a clear advantage of any single mAb.

SF36 improvements over follow-up were similar in participants Under and Over 50 (no overall interaction between Age and Time). Compared with baseline, a significant decline in SF36 scores was observed only at 6 months (*β* = −10.42, *p* = 0.002). Regarding treatment effects, both galcanezumab and fremanezumab showed significant positive interactions at 6 months (*β* = 13.47, *p* = 0.001 and *β* = 12.42, *p* = 0.003, respectively), suggesting transient mid-term improvements.

MIDAS scores declined dramatically (from −56.85, 95% CI −67.90 to −45.79, at T3 to −64.39, 95% CI −76.07 to −52.71, points at T6; all *p* < 0.001), indicating substantial improvement in migraine-related disability. Age did not influence baseline disability and no significant baseline or longitudinal treatment differences were observed, demonstrating that improvements in disability were broadly comparable across all medications.

For HIT-6, negative coefficients indicate greater improvement (larger score reductions), whereas positive coefficients indicate smaller improvements relative to the reference treatment (erenumab). HIT-6 scores decreased significantly at all follow-up visits, from −9.34 points (95% CI − 12.14 to −6.54) at T3 to −11.63 points (95% CI − 14.53 to −8.73) at T6 (all *p* < 0.001), indicating a clinically meaningful improvement in headache-related impact. Age was not significantly associated with HIT-6 change. Compared with erenumab, galcanezumab was associated with a greater reduction in HIT-6 scores at 12 months (additional reduction of 4.27 points; *p* = 0.014). In contrast, eptinezumab showed smaller reductions in HIT-6 scores during the early follow-up period, with improvements that were 6.36 points smaller at 3 months (*p* = 0.001) and 8.95 points smaller at 6 months (*p* < 0.001) relative to erenumab.

BDI scores decreased meaningfully over time (−4.12, 95% CI −6.33 to −1.90, points at T3 to −6.06, 95% CI −8.44 to −3.69, points at T6 in the BDI total score; all *p* ≤ 0.002). Older adults had lower baseline depression scores (−2.46, *p* = 0.014), but longitudinal trajectories did not differ by age. No significant baseline differences were detected across treatments. At 6 months, galcanezumab (2.94, *p* = 0.03), fremanezumab (3.51, *p* = 0.016) and eptinezumab (4.58, *p* = 0.005) showed additional improvements relative to erenumab, but no differences appeared at the other timepoints.

Anxiety symptoms decreased modestly early on (−2.77, 95% CI −4.49 to −1.05, *p* = 0.002 in the Zung score at 3 months), maintaining the same trend at 6 months (−2.19, 95% CI −4.45 to 0.07, *p* = 0.06 in the Zung score), with no sustained improvement at 12 months. Older adults had lower baseline anxiety (−2.37, *p* = 0.001), but age did not shape longitudinal change. No treatment group differed at baseline or over time, indicating similar anxiety trajectories across all medications.

Sensitivity analyses restricted to participants with complete follow-up data yielded results consistent with the primary analyses (Supplementary Table 4).

### Prediction of treatment response

**Figure 3:**
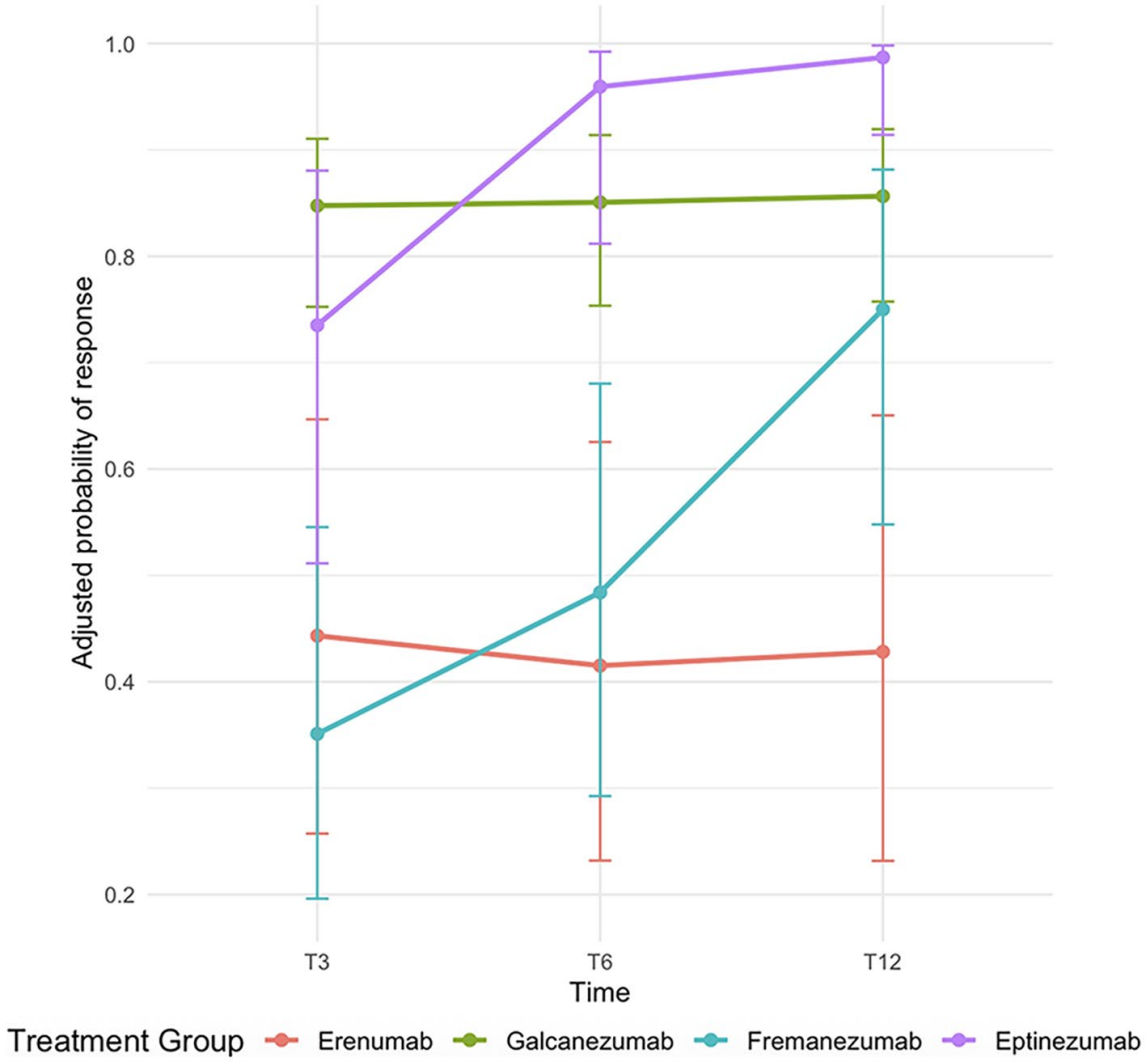
Probability of response over time across different treatment groups.

The random intercept variance in the final GLMM (σ^2^ = 2.43, SD = 1.56) indicated substantial heterogeneity in individual baseline response probabilities (Figure 3; Supplementary Table 5). After adjustment for dose variation and age, the overall probability of being a responder did not differ significantly between erenumab and fremanezumab or eptinezumab at the early visits (T3–T6). At 12 months, patients treated with fremanezumab or eptinezumab were significantly more likely to achieve a ≥ 50% reduction in monthly migraine days than those treated with erenumab. This advantage emerged specifically at the 12-month follow-up (fremanezumab × T12: *β* = 1.78, SE = 0.59, *p* = 0.003; eptinezumab × T12: *β* = 3.36, SE = 0.93, *p* < 0.001). In addition, galcanezumab was associated with a higher likelihood of achieving a ≥ 50% response overall compared with erenumab, regardless of follow-up time (*β* = 1.94, SE = 0.61, *p* = 0.001). Age group was not significantly associated with treatment response.

The dose-adjusted covariate was significantly associated with responder status (*β* = −0.012, SE = 0.004, *p* = 0.001). Higher administered doses were associated with slightly lower odds of response. This finding may reflect confounding by indication, whereby patients requiring higher doses had more refractory disease.

Results were unchanged when age was modeled as a continuous variable instead of using age groups, indicating that the findings were robust to the method used to account for age (Supplementary Table 6). The sensitivity analysis with additional covariates (Supplementary Table 7) gave the same results.

In the ≥65 subgroup, compared to the probability of response at 3 months, the odds of response were higher at 12 months (*β* = 0.685, SE = 0.203, *p* < 0.001), whereas the increase at 6 months was not statistically significant. There was no evidence that the change in response over time differed significantly between patients over 65 and younger patients, as neither the 6-month interaction nor the 12-month interaction was significant. Overall, these exploratory findings seem to suggest an improvement in responder probability over time, particularly by 12 months, but do not support a clear modifying effect of being over 65 years old on treatment response trajectories (Supplementary Table 8).

### Patterns of clinical outcomes

To explore the shared variance among changes in clinical outcomes over time, we performed a PCA on the delta scores of MIDAS, BDI, HIT-6, Zung, and SF36 at T3, T6, and T12. The Kaiser–Meyer–Olkin measure of sampling adequacy was 0.61, suggesting sufficient adequacy for PCA. Two components (PC1 and PC2) were retained (Supplementary Figure 1; Supplementary Table 9).

**Figure 4:**
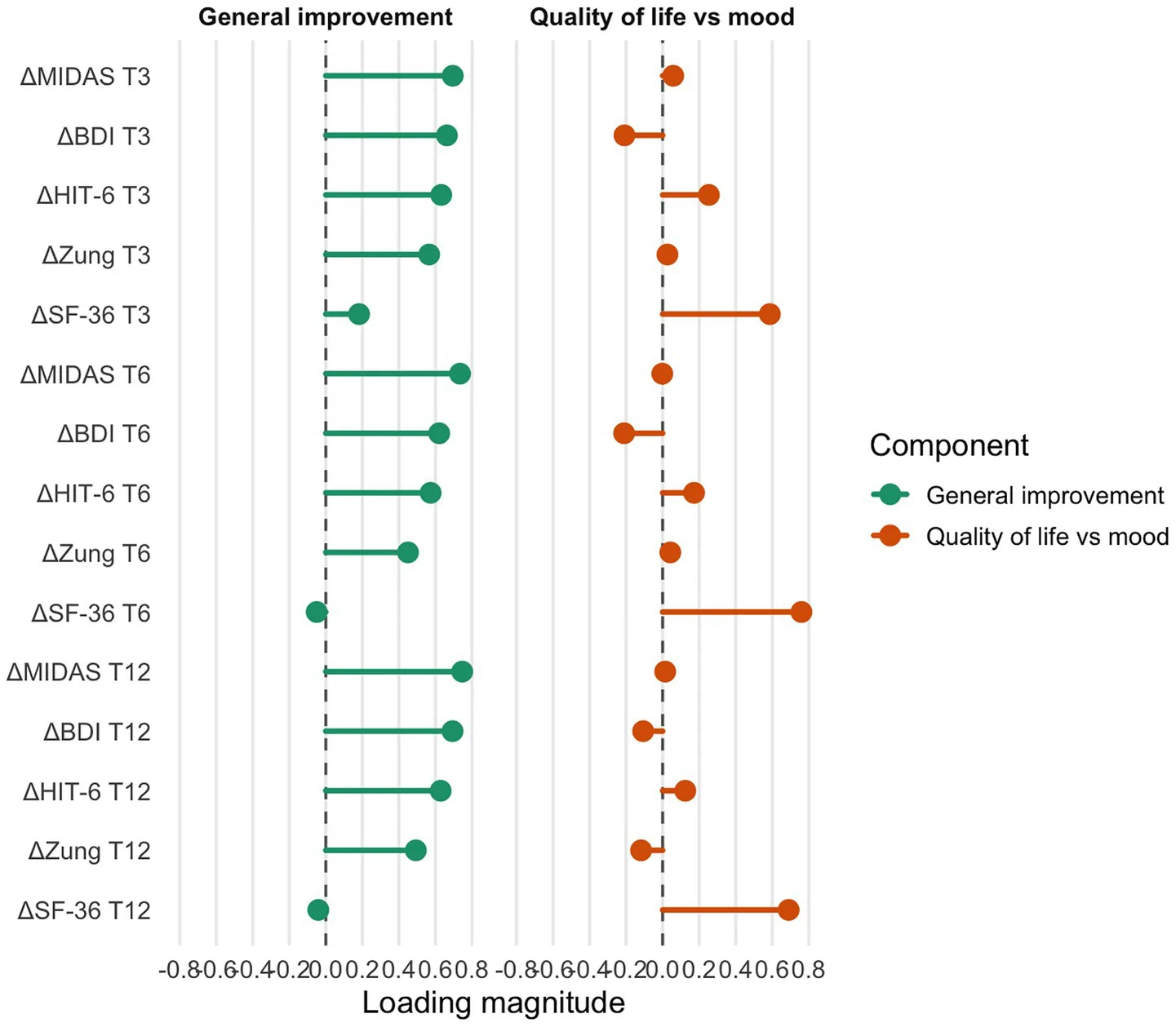
Component loadings from principal component analysis. BDI, Beck Depression Inventory; HIT-6, Headache Impact Test-6; MIDAS, Migraine Disability Assessment; SF36, Short Form 36.

PC1, which can be described as *General improvement*, explained 30.5% of the variance and was primarily defined by changes in migraine-related disability (ΔMIDAS), depression (ΔBDI), headache impact (ΔHIT-6), and anxiety (ΔZung) across all timepoints, with loadings ranging from 0.410 to 0.731. PC1 thus represents a multidomain improvement across migraine-related disability, headache impact, depression, and anxiety. PC2, renamed *Quality of life versus mood*, explained 11.3% of the variance and was mainly associated with changes in quality of life (ΔSF36) at T3, T6, and T12 (loadings 0.576–0.655), with minor opposite sign contributions from BDI at T3, T6 and T12 (loadings −0.322 – −0.243) and from Zung at T12 (loading −0.285) (Figure 4). Loadings of opposite signs for quality-of-life and mood-related measures contribute in contrasting directions to the latent dimension highlighted by PC2, indicating that improvements in health-related quality of life were only partially aligned with changes in mood.

PC1 *General improvement* differed significantly across Treatment types [χ^2^(3) = 12.91, *p* = 0.005], but not age groups. *Post hoc* Dunn tests with Bonferroni correction suggested that patients treated with eptinezumab could have significantly higher PC1 scores compared with those receiving treated with erenumab (*p* = 0.01), fremanezumab (*p* = 0.04), and galcanezumab (*p* = 0.003), reflecting a more pronounced multidomain clinical improvement (Supplementary Figure 2).

PC2 *Quality of life versus mood* differed significantly across Treatment types [χ^2^(3) = 8.97, *p* = 0.03], but not Age groups. Dunn’s *post-hoc* tests with Bonferroni correction appeared to indicate a significant difference in PC2 scores between the eptinezumab and galcanezumab groups (*p* = 0.017), with no other significant pairwise differences observed (Supplementary Figure 3).

## Discussion

In this retrospective, single center, real-world cohort, treatment with CGRP mAbs was associated with rapid, substantial, and broadly sustained clinical improvements across a wide range of migraine-related and patient-reported outcomes over 12 months.

Age did not significantly shape any outcome at baseline, nor did it modify treatment response throughout the 12-month period in our linear mixed effect models. Also, age was not associated with treatment success, indicating comparable response patterns across younger and older adults. This contrasts with the large study by Caronna et al. (10), in which older age predicted ≥50% MMD reduction. Their much larger sample, lack of eptinezumab-treated participants, and analysis restricted to six months may partly explain the difference.

Among the limited number of studies that specifically examined the truly older population (≥65 years) (16), Cetta et al. (17) studied 15 older adults treated with erenumab and reported significant within-group improvements, but no differences between older and younger patients. In another study in patients over 65 years, CGRP mAbs were safe and effective in real-life clinical practice, with fremanezumab showing fewer adverse effects (16). In addition to our primary age with an under/over-50 threshold, our exploratory analysis of participants aged ≥65 suggested improvements over time without significant divergence from younger ones. Given the limited sample size, this analysis was intended to be descriptive and hypothesis-generating rather than confirmatory. Our findings could imply that older adults may derive clear clinical benefits from CGRP-mAbs; however, whether age alone meaningfully modifies treatment response remains debatable. Larger studies with more granular age stratification (e.g., ≥70 or even ≥80 years) are needed to elucidate potential age-related differences and the possible effect of comorbidities and frailty status.

Methodologically, the present study uses statistically powerful and rigorous longitudinal models to capture the heterogeneity and temporal dynamics of migraine outcomes, aligning with the methodological perspective put forward by Di Tanna et al. (31). In our linear mixed model, the reductions in MMD were both clinically meaningful and temporally stable, with the largest gains occurring in the first three months and persisting through one year. The magnitude of improvement observed is consistent with previously reported responder rates (32, 33) and is coherent with the notion that CGRP inhibition produces an early and durable preventive effect (34, 35). Small baseline differences between treatment groups—such as higher MMD and acute medication consumption in the eptinezumab group—did not translate into consistent longitudinal differences. Although fremanezumab showed a slightly attenuated early response and eptinezumab trended toward greater benefit at 12 months, these effects were transient or not robust to conservative variance estimation, underscoring the overall similarity of treatment trajectories. Acute medication consumption showed an even more pronounced decline than MMD, with reductions exceeding nine days per month at all follow-up visits. This suggests a meaningful shift away from medication overuse patterns, an important clinical target given its tight link to chronification.

We observed substantial variability in baseline response probabilities, but early treatment effects (T3–T6) were similar across erenumab, fremanezumab, and eptinezumab. Our T12 outcomes mirror the sustained one-year improvements reported in clinical trials and real-world cohorts, including reductions in migraine frequency and functional burden seen with erenumab (36), high likelihood of continued response (37) and sustained multidomain benefits with eptinezumab (38, 39).

Incorporating dose adjustments for galcanezumab and eptinezumab allowed us to account for loading doses and escalation strategies, and the small inverse association between administered dose and response likely reflects higher doses being used in more refractory patients for eptinezumab. By 12 months, fremanezumab and eptinezumab showed higher odds of achieving a ≥ 50% response compared with erenumab, while galcanezumab demonstrated consistently greater odds independent of time, even after the post-loading dose reduction. Galcanezumab effect on “whole pain burden” and multidimensional outcomes in migraine patients had already been demonstrated in a real world cohort of migranous patients experiencing previous unsuccessful preventive treatments (40). Regarding eptinezumab, the capacity to switch or escalate to this agent may represent an important opportunity for patients who do not rapidly respond sufficiently. The favorable long-term profile of eptinezumab shown in DELIVER (38) suggests that incorporating it earlier, or as an option for non-responders, could help address the substantial residual burden still evident in many real-world populations like ours.

Pain intensity, disability (MIDAS), and headache-related impact (HIT-6) all demonstrated significant and sustained improvements with CGRP mAbs. HIT-6 trajectories revealed somewhat more pronounced early benefit in the eptinezumab group; however, treatment differences were not consistent over time, again indicating broadly comparable long-term effects across mAbs. In our linear mixed effect models, psychological symptoms also improved, particularly depressive symptoms, which showed the greatest reduction at mid-treatment. Anxiety levels decreased modestly early on but did not maintain significance at 12 months. Notably, older adults exhibited lower baseline depression and anxiety, but neither domain showed age-related differences in treatment response, reinforcing the generalizability of clinical benefit across age groups, irrespective of the presence of comorbid mood-related symptoms (41). At 6 months, galcanezumab, fremanezumab and eptinezumab showed additional improvements in BDI scores: this had already been shown for fremanezumab (42–45), not yet for galcanezumab and eptinezumab.

Even if modeled longitudinally, response trajectories could not add information which we could instead observe in clinical practice. The PCA of changes in clinical outcomes may indicate two distinct dimensions underlying patients’ longitudinal improvement with CGRP mAbs. The first component, *General improvement*, captured broad reductions in migraine-related disability, headache impact, depressive symptoms, and anxiety. This multidomain component appear to indicate the interconnected nature of migraine burden and psychological distress and may reflect the perceived effectiveness of the preventive therapy, which contributes to greater patient engagement. The second component, *Quality of life versus mood*, is compatible with a separate dimension in which improvements in health-related quality of life were only partially aligned with changes in mood, emphasizing that quality-of-life gains may occur through mechanisms not directly tied to emotional symptoms.

Importantly, *General improvement* seemed to significantly across treatments, with eptinezumab demonstrating greater multidomain benefit compared with other mAbs. This pattern could be consistent with the more pronounced clinical response observed for eptinezumab at later time points in our mixed-effects analyses and may also reflect a more favorable evaluation of treatment response among patients who initiated therapy with a higher baseline disease burden. Additionally, these findings may be partly explained by differences in pharmacokinetic profiles across mAbs, as eptinezumab is administered intravenously and achieves therapeutic concentrations more rapidly, potentially leading to earlier clinical benefit. In contrast, the *Quality of life versus mood* component appeared to remain stable across age and groups, with a small difference highlighted only for eptinezumab compared to galcanezumab. This consistency across mAbs may reflect patient-specific factors [e.g., different personality traits (46)] rather than medication-specific effects.

The multidimensional pattern emerging from our exploratory PCA may align closely with evidence from randomized controlled trials. In the REGAIN study of galcanezumab for CM, patients experienced parallel improvements across disability (MIDAS) and health-related quality of life domains (47). Notably, REGAIN also showed that improvements in quality of life were not always proportional to MIDAS reductions, suggesting partly independent pathways of recovery. Our PCA seemed to extend these findings by statistically decomposing this multidimensional change into its underlying structure.

Prior real-world studies (48) demonstrated that responders to CGRP mAbs experienced concurrent improvements in disability, headache impact, mood symptoms, and quality of life, paralleling the broad *General improvement* dimension. In those studies, quality of life measures improved even in partial responders, whereas mood scores did not, aligning with our PC2 component, which separates quality-of-life gains from affective improvement.

This study has several limitations. First, it was conducted at a single center and included a relatively small cohort, with missing data at long-term follow-up visits and an even smaller subgroup treated with eptinezumab, the most recently approved CGRP mAb. The statistical approach was adapted to account for the limited sample size, which may nonetheless affect the robustness of the findings. Second, the reliance on self-reported outcomes represents an additional important limitation. Third, a control group receiving traditional oral prophylaxis was not included, as such patients typically lack regular follow-up visits and consistent documentation of migraine burden. Fourth, to address confounding by indication, we performed additional analyses adjusting for available baseline clinical characteristics. Nevertheless, because treatment allocation was not randomized and some potentially relevant factors may not have been fully captured, residual confounding cannot be excluded and should be considered when interpreting treatment-group comparisons. Fifth, variable outcome availability across time points precluded a clinically meaningful distinction between who did and did not complete one year of treatment with CGRP mAbs, therefore baseline comparisons by completion status were not performed. Finally, psychiatric comorbidities and related therapies were not systematically recorded, potentially influencing observed mood variations.

In conclusion, in this real-world, longitudinal study, overall effectiveness of CGRP mAbs was comparable in a 12-month follow-up, with only modest and time-dependent differences emerging between agents. Age did not meaningfully influence baseline disease burden or treatment response, and both younger and older adults derived similar clinical benefit, supporting the use of CGRP mAbs across a wide age spectrum.

Beyond traditional clinical endpoints, a multidimensional analysis of longitudinal changes revealed the interconnected improvement of migraine burden, mood, and disability. In this context, eptinezumab seemed to be associated with greater multidomain benefit compared with some subcutaneous agents, a finding that may reflect differences in baseline disease severity, patient-perceived effectiveness, and pharmacokinetic properties related to intravenous administration. These multidomain effects underscore the importance of considering patient-reported outcomes and composite measures when evaluating preventive migraine therapies.

Taken together, our findings reinforce the real-world effectiveness of CGRP mAbs as long-term preventive treatments for migraine and suggest that treatment choice may be guided not only by reductions in MMD, but also by broader patterns of functional and psychological improvement. Larger, multicenter studies with longer follow-up and more granular age stratification are warranted to confirm these observations.

## Data Availability

The raw data supporting the conclusions of this article will be made available by the authors, upon motivated request.
