# Supplementary material for: Tracking migraine outcomes over time by age and monoclonal antibody type: a retrospective, single-center, real-world study
Source: Front Neurol. 2026 Aug 11;17:1893551. doi: 10.3389/fneur.2026.1893551 (PMC13521433; doi:10.3389/fneur.2026.1893551)
Supplement: Supplementary file 1 [file Data_Sheet_1.docx]

Supplementary Table 1: number of available observations for each outcome at each assessment time point

|  | T0 | T3 | T6 | T12 |
| --- | --- | --- | --- | --- |
| MMD | 450 | 413 | 379 | 319 |
| Monthly acute medication intake | 440 | 412 | 376 | 317 |
| NRS | 443 | 411 | 374 | 316 |
| MIDAS | 441 | 364 | 327 | 290 |
| HIT-6 | 411 | 338 | 310 | 258 |
| SF36 | 415 | 338 | 312 | 263 |
| BDI | 419 | 340 | 309 | 263 |
| ZUNG | 421 | 341 | 306 | 263 |

Legend: BDI= Beck Depression Inventory; HIT-6=Headache Impact Test-6; MIDAS= Migraine Disability Assessment; MMD= Monthly Migraine Days; NRS= Numerical Rating Scale; SF36= Short Form 36

Supplementary Table 2: patients with a previous anti-CGRP treatment

| Erenumab | 0/107 |
| --- | --- |
| Galcanezumab | 7/180 (7 erenumab) |
| Fremanezumab | 2/110 (2 erenumab) |
| Eptinezumab | 19/63 (10 erenumab, 6 galcanezumab, 3 fremanezumab) |

Supplementary Table 3: linear mixed model results

| Outcome | Term | Estimate | Std.error | Conf.low | Conf.high | Statistic | p.value |
| --- | --- | --- | --- | --- | --- | --- | --- |
| MMD | (Intercept) | 18.210 | 0.783 | 16.673 | 19.746 | 23.250 | 0.000 |
|  | TimeT3 | -9.870 | 0.774 | -11.389 | -8.351 | -12.749 | 0.000 |
|  | TimeT6 | -8.785 | 0.805 | -10.365 | -7.205 | -10.908 | 0.000 |
|  | TimeT12 | -9.135 | 0.878 | -10.859 | -7.412 | -10.399 | 0.000 |
|  | Age_groupOver 50 | 0.322 | 0.723 | -1.098 | 1.742 | 0.446 | 0.656 |
|  | Treatment_groupGalcanezumab | -1.728 | 0.909 | -3.514 | 0.058 | -1.902 | 0.058 |
|  | Treatment_groupFremanezumab | -0.447 | 1.003 | -2.419 | 1.525 | -0.445 | 0.656 |
|  | Treatment_groupEptinezumab | 3.961 | 1.219 | 1.566 | 6.357 | 3.250 | 0.001 |
|  | TimeT3:Age_groupOver 50 | -0.537 | 0.706 | -1.922 | 0.847 | -0.761 | 0.447 |
|  | TimeT6:Age_groupOver 50 | -0.967 | 0.728 | -2.397 | 0.462 | -1.328 | 0.184 |
|  | TimeT12:Age_groupOver 50 | -0.075 | 0.770 | -1.586 | 1.435 | -0.098 | 0.922 |
|  | TimeT3:Treatment_groupGalcanezumab | 1.012 | 0.904 | -0.761 | 2.785 | 1.120 | 0.263 |
|  | TimeT6:Treatment_groupGalcanezumab | -0.825 | 0.939 | -2.666 | 1.017 | -0.879 | 0.380 |
|  | TimeT12:Treatment_groupGalcanezumab | -0.188 | 1.010 | -2.170 | 1.793 | -0.186 | 0.852 |
|  | TimeT3:Treatment_groupFremanezumab | 2.318 | 0.986 | 0.384 | 4.252 | 2.352 | 0.019 |
|  | TimeT6:Treatment_groupFremanezumab | -0.379 | 1.020 | -2.380 | 1.623 | -0.371 | 0.710 |
|  | TimeT12:Treatment_groupFremanezumab | -2.148 | 1.082 | -4.271 | -0.024 | -1.985 | 0.047 |
|  | TimeT3:Treatment_groupEptinezumab | 0.579 | 1.177 | -1.730 | 2.889 | 0.492 | 0.623 |
|  | TimeT6:Treatment_groupEptinezumab | -0.948 | 1.205 | -3.312 | 1.416 | -0.787 | 0.432 |
|  | TimeT12:Treatment_groupEptinezumab | -5.676 | 1.348 | -8.322 | -3.030 | -4.209 | 0.000 |
| Monthly acute medications | (Intercept) | 17.820 | 1.291 | 15.287 | 20.353 | 13.804 | 0.000 |
|  | TimeT3 | -11.346 | 1.407 | -14.106 | -8.586 | -8.067 | 0.000 |
|  | TimeT6 | -9.827 | 1.484 | -12.738 | -6.915 | -6.622 | 0.000 |
|  | TimeT12 | -10.370 | 1.597 | -13.503 | -7.236 | -6.493 | 0.000 |
|  | Age_groupOver 50 | 0.663 | 1.186 | -1.668 | 2.995 | 0.559 | 0.576 |
|  | Treatment_groupGalcanezumab | 1.009 | 1.495 | -1.929 | 3.947 | 0.675 | 0.500 |
|  | Treatment_groupFremanezumab | -1.237 | 1.648 | -4.476 | 2.001 | -0.751 | 0.453 |
|  | Treatment_groupEptinezumab | 8.318 | 1.988 | 4.410 | 12.226 | 4.183 | 0.000 |
|  | TimeT3:Age_groupOver 50 | -0.235 | 1.284 | -2.755 | 2.284 | -0.183 | 0.855 |
|  | TimeT6:Age_groupOver 50 | 0.064 | 1.327 | -2.541 | 2.668 | 0.048 | 0.962 |
|  | TimeT12:Age_groupOver 50 | -0.263 | 1.401 | -3.011 | 2.486 | -0.188 | 0.851 |
|  | TimeT3:Treatment_groupGalcanezumab | -1.380 | 1.642 | -4.603 | 1.842 | -0.841 | 0.401 |
|  | TimeT6:Treatment_groupGalcanezumab | -2.568 | 1.720 | -5.942 | 0.806 | -1.493 | 0.136 |
|  | TimeT12:Treatment_groupGalcanezumab | -2.111 | 1.838 | -5.718 | 1.495 | -1.149 | 0.251 |
|  | TimeT3:Treatment_groupFremanezumab | 2.234 | 1.794 | -1.287 | 5.755 | 1.245 | 0.213 |
|  | TimeT6:Treatment_groupFremanezumab | -0.143 | 1.869 | -3.810 | 3.524 | -0.077 | 0.939 |
|  | TimeT12:Treatment_groupFremanezumab | -0.816 | 1.973 | -4.688 | 3.057 | -0.413 | 0.679 |
|  | TimeT3:Treatment_groupEptinezumab | -1.606 | 2.135 | -5.795 | 2.584 | -0.752 | 0.452 |
|  | TimeT6:Treatment_groupEptinezumab | -2.015 | 2.196 | -6.324 | 2.293 | -0.918 | 0.359 |
|  | TimeT12:Treatment_groupEptinezumab | -1.324 | 2.445 | -6.121 | 3.473 | -0.542 | 0.588 |
| NRS | (Intercept) | 7.998 | 0.107 | 7.788 | 8.208 | 74.738 | 0.000 |
|  | TimeT3 | -1.275 | 0.175 | -1.620 | -0.931 | -7.270 | 0.000 |
|  | TimeT6 | -1.568 | 0.195 | -1.951 | -1.186 | -8.040 | 0.000 |
|  | TimeT12 | -1.581 | 0.242 | -2.055 | -1.106 | -6.535 | 0.000 |
|  | Age_groupOver 50 | 0.005 | 0.101 | -0.194 | 0.203 | 0.049 | 0.961 |
|  | Treatment_groupGalcanezumab | -0.214 | 0.126 | -0.462 | 0.033 | -1.705 | 0.089 |
|  | Treatment_groupFremanezumab | -0.220 | 0.139 | -0.493 | 0.054 | -1.580 | 0.115 |
|  | Treatment_groupEptinezumab | -0.151 | 0.168 | -0.481 | 0.179 | -0.901 | 0.368 |
|  | TimeT3:Age_groupOver 50 | -0.239 | 0.184 | -0.600 | 0.123 | -1.295 | 0.196 |
|  | TimeT6:Age_groupOver 50 | 0.042 | 0.186 | -0.322 | 0.407 | 0.228 | 0.819 |
|  | TimeT12:Age_groupOver 50 | 0.281 | 0.226 | -0.162 | 0.724 | 1.245 | 0.213 |
|  | TimeT3:Treatment_groupGalcanezumab | -0.703 | 0.231 | -1.156 | -0.251 | -3.048 | 0.002 |
|  | TimeT6:Treatment_groupGalcanezumab | -0.306 | 0.239 | -0.776 | 0.164 | -1.279 | 0.201 |
|  | TimeT12:Treatment_groupGalcanezumab | -0.531 | 0.288 | -1.096 | 0.034 | -1.845 | 0.065 |
|  | TimeT3:Treatment_groupFremanezumab | -0.238 | 0.240 | -0.708 | 0.232 | -0.993 | 0.321 |
|  | TimeT6:Treatment_groupFremanezumab | -0.138 | 0.256 | -0.640 | 0.365 | -0.538 | 0.591 |
|  | TimeT12:Treatment_groupFremanezumab | -0.632 | 0.315 | -1.251 | -0.014 | -2.005 | 0.045 |
|  | TimeT3:Treatment_groupEptinezumab | 0.125 | 0.267 | -0.400 | 0.649 | 0.466 | 0.641 |
|  | TimeT6:Treatment_groupEptinezumab | 0.273 | 0.280 | -0.277 | 0.823 | 0.974 | 0.330 |
|  | TimeT12:Treatment_groupEptinezumab | -0.065 | 0.359 | -0.769 | 0.638 | -0.183 | 0.855 |
| SF36 | (Intercept) | 106.291 | 2.145 | 102.081 | 110.501 | 49.556 | 0.000 |
|  | TimeT3 | -3.290 | 3.295 | -9.758 | 3.178 | -0.998 | 0.318 |
|  | TimeT6 | -10.419 | 3.397 | -17.086 | -3.753 | -3.068 | 0.002 |
|  | TimeT12 | -1.157 | 3.565 | -8.155 | 5.840 | -0.325 | 0.746 |
|  | Age_groupOver 50 | -2.935 | 1.926 | -6.721 | 0.851 | -1.524 | 0.128 |
|  | Treatment_groupGalcanezumab | -4.111 | 2.472 | -8.969 | 0.746 | -1.663 | 0.097 |
|  | Treatment_groupFremanezumab | -1.697 | 2.706 | -7.015 | 3.621 | -0.627 | 0.531 |
|  | Treatment_groupEptinezumab | 0.714 | 3.259 | -5.691 | 7.120 | 0.219 | 0.827 |
|  | TimeT3:Age_groupOver 50 | 4.600 | 2.804 | -0.903 | 10.104 | 1.641 | 0.101 |
|  | TimeT6:Age_groupOver 50 | 0.063 | 2.867 | -5.563 | 5.690 | 0.022 | 0.982 |
|  | TimeT12:Age_groupOver 50 | 5.921 | 3.010 | 0.012 | 11.829 | 1.967 | 0.050 |
|  | TimeT3:Treatment_groupGalcanezumab | 1.676 | 3.791 | -5.765 | 9.116 | 0.442 | 0.659 |
|  | TimeT6:Treatment_groupGalcanezumab | 13.469 | 3.921 | 5.775 | 21.164 | 3.436 | 0.001 |
|  | TimeT12:Treatment_groupGalcanezumab | 0.007 | 4.062 | -7.967 | 7.980 | 0.002 | 0.999 |
|  | TimeT3:Treatment_groupFremanezumab | 1.613 | 4.105 | -6.444 | 9.669 | 0.393 | 0.695 |
|  | TimeT6:Treatment_groupFremanezumab | 12.423 | 4.167 | 4.245 | 20.602 | 2.981 | 0.003 |
|  | TimeT12:Treatment_groupFremanezumab | -2.783 | 4.346 | -11.313 | 5.748 | -0.640 | 0.522 |
|  | TimeT3:Treatment_groupEptinezumab | -0.050 | 4.746 | -9.365 | 9.266 | -0.010 | 0.992 |
|  | TimeT6:Treatment_groupEptinezumab | 10.497 | 4.785 | 1.105 | 19.889 | 2.194 | 0.029 |
|  | TimeT12:Treatment_groupEptinezumab | -2.400 | 5.280 | -12.762 | 7.963 | -0.455 | 0.650 |
| MIDAS | (Intercept) | 87.613 | 4.590 | 78.606 | 96.620 | 19.089 | 0.000 |
|  | TimeT3 | -56.845 | 5.635 | -67.904 | -45.786 | -10.087 | 0.000 |
|  | TimeT6 | -64.388 | 5.952 | -76.069 | -52.706 | -10.817 | 0.000 |
|  | TimeT12 | -60.152 | 6.209 | -72.338 | -47.967 | -9.688 | 0.000 |
|  | Age_groupOver 50 | 2.395 | 4.206 | -5.871 | 10.660 | 0.569 | 0.569 |
|  | Treatment_groupGalcanezumab | -0.496 | 5.316 | -10.943 | 9.951 | -0.093 | 0.926 |
|  | Treatment_groupFremanezumab | -2.689 | 5.851 | -14.188 | 8.811 | -0.459 | 0.646 |
|  | Treatment_groupEptinezumab | 4.460 | 7.064 | -9.422 | 18.343 | 0.631 | 0.528 |
|  | TimeT3:Age_groupOver 50 | -8.276 | 4.870 | -17.833 | 1.280 | -1.700 | 0.090 |
|  | TimeT6:Age_groupOver 50 | -4.325 | 5.034 | -14.203 | 5.554 | -0.859 | 0.390 |
|  | TimeT12:Age_groupOver 50 | -3.171 | 5.215 | -13.404 | 7.063 | -0.608 | 0.543 |
|  | TimeT3:Treatment_groupGalcanezumab | -4.774 | 6.507 | -17.543 | 7.996 | -0.734 | 0.463 |
|  | TimeT6:Treatment_groupGalcanezumab | -4.716 | 6.863 | -18.184 | 8.753 | -0.687 | 0.492 |
|  | TimeT12:Treatment_groupGalcanezumab | -6.438 | 7.038 | -20.249 | 7.374 | -0.915 | 0.361 |
|  | TimeT3:Treatment_groupFremanezumab | 4.259 | 7.048 | -9.572 | 18.091 | 0.604 | 0.546 |
|  | TimeT6:Treatment_groupFremanezumab | 3.093 | 7.296 | -11.225 | 17.411 | 0.424 | 0.672 |
|  | TimeT12:Treatment_groupFremanezumab | -2.908 | 7.561 | -17.746 | 11.930 | -0.385 | 0.701 |
|  | TimeT3:Treatment_groupEptinezumab | 11.234 | 8.158 | -4.776 | 27.245 | 1.377 | 0.169 |
|  | TimeT6:Treatment_groupEptinezumab | 13.734 | 8.365 | -2.681 | 30.150 | 1.642 | 0.101 |
|  | TimeT12:Treatment_groupEptinezumab | 3.855 | 9.109 | -14.022 | 21.731 | 0.423 | 0.672 |
| HIT-6 | (Intercept) | 66.196 | 1.051 | 64.134 | 68.258 | 63.004 | 0.000 |
|  | TimeT3 | -9.341 | 1.427 | -12.142 | -6.540 | -6.546 | 0.000 |
|  | TimeT6 | -11.630 | 1.480 | -14.534 | -8.726 | -7.860 | 0.000 |
|  | TimeT12 | -7.260 | 1.548 | -10.298 | -4.222 | -4.690 | 0.000 |
|  | Age_groupOver 50 | -0.768 | 0.892 | -2.521 | 0.985 | -0.861 | 0.390 |
|  | Treatment_groupGalcanezumab | -1.058 | 1.184 | -3.386 | 1.269 | -0.894 | 0.372 |
|  | Treatment_groupFremanezumab | -0.691 | 1.285 | -3.216 | 1.833 | -0.538 | 0.591 |
|  | Treatment_groupEptinezumab | -2.848 | 1.531 | -5.858 | 0.161 | -1.860 | 0.064 |
|  | TimeT3:Age_groupOver 50 | 0.057 | 1.146 | -2.193 | 2.307 | 0.050 | 0.960 |
|  | TimeT6:Age_groupOver 50 | -0.115 | 1.179 | -2.429 | 2.200 | -0.097 | 0.923 |
|  | TimeT12:Age_groupOver 50 | 0.855 | 1.251 | -1.601 | 3.311 | 0.683 | 0.495 |
|  | TimeT3:Treatment_groupGalcanezumab | 0.461 | 1.617 | -2.711 | 3.634 | 0.285 | 0.775 |
|  | TimeT6:Treatment_groupGalcanezumab | 2.108 | 1.680 | -1.190 | 5.405 | 1.254 | 0.210 |
|  | TimeT12:Treatment_groupGalcanezumab | -4.274 | 1.733 | -7.675 | -0.872 | -2.466 | 0.014 |
|  | TimeT3:Treatment_groupFremanezumab | 2.239 | 1.734 | -1.164 | 5.641 | 1.291 | 0.197 |
|  | TimeT6:Treatment_groupFremanezumab | 0.246 | 1.765 | -3.218 | 3.711 | 0.140 | 0.889 |
|  | TimeT12:Treatment_groupFremanezumab | -2.726 | 1.857 | -6.370 | 0.918 | -1.468 | 0.142 |
|  | TimeT3:Treatment_groupEptinezumab | 6.357 | 1.989 | 2.453 | 10.261 | 3.196 | 0.001 |
|  | TimeT6:Treatment_groupEptinezumab | 8.945 | 2.009 | 5.001 | 12.888 | 4.452 | 0.000 |
|  | TimeT12:Treatment_groupEptinezumab | 2.572 | 2.228 | -1.800 | 6.944 | 1.155 | 0.249 |
| BDI | (Intercept) | 14.800 | 1.101 | 12.640 | 16.960 | 13.447 | 0.000 |
|  | TimeT3 | -4.115 | 1.127 | -6.326 | -1.903 | -3.652 | 0.000 |
|  | TimeT6 | -6.063 | 1.210 | -8.437 | -3.689 | -5.012 | 0.000 |
|  | TimeT12 | -3.875 | 1.248 | -6.323 | -1.426 | -3.106 | 0.002 |
|  | Age_groupOver 50 | -2.462 | 0.997 | -4.421 | -0.502 | -2.469 | 0.014 |
|  | Treatment_groupGalcanezumab | -1.191 | 1.271 | -3.690 | 1.308 | -0.937 | 0.349 |
|  | Treatment_groupFremanezumab | 0.170 | 1.394 | -2.570 | 2.910 | 0.122 | 0.903 |
|  | Treatment_groupEptinezumab | 2.589 | 1.691 | -0.734 | 5.913 | 1.531 | 0.126 |
|  | TimeT3:Age_groupOver 50 | 0.078 | 0.926 | -1.739 | 1.895 | 0.084 | 0.933 |
|  | TimeT6:Age_groupOver 50 | 0.947 | 0.958 | -0.933 | 2.828 | 0.989 | 0.323 |
|  | TimeT12:Age_groupOver 50 | 1.719 | 1.011 | -0.265 | 3.704 | 1.700 | 0.089 |
|  | TimeT3:Treatment_groupGalcanezumab | 1.934 | 1.291 | -0.600 | 4.467 | 1.498 | 0.135 |
|  | TimeT6:Treatment_groupGalcanezumab | 2.939 | 1.378 | 0.234 | 5.644 | 2.133 | 0.033 |
|  | TimeT12:Treatment_groupGalcanezumab | 1.404 | 1.411 | -1.364 | 4.173 | 0.995 | 0.320 |
|  | TimeT3:Treatment_groupFremanezumab | 2.145 | 1.386 | -0.575 | 4.865 | 1.548 | 0.122 |
|  | TimeT6:Treatment_groupFremanezumab | 3.506 | 1.449 | 0.663 | 6.349 | 2.420 | 0.016 |
|  | TimeT12:Treatment_groupFremanezumab | -0.071 | 1.507 | -3.029 | 2.888 | -0.047 | 0.963 |
|  | TimeT3:Treatment_groupEptinezumab | 2.250 | 1.593 | -0.878 | 5.377 | 1.412 | 0.158 |
|  | TimeT6:Treatment_groupEptinezumab | 4.575 | 1.640 | 1.356 | 7.794 | 2.789 | 0.005 |
|  | TimeT12:Treatment_groupEptinezumab | 2.272 | 1.792 | -1.245 | 5.789 | 1.268 | 0.205 |
| ZUNG | (Intercept) | 38.305 | 0.792 | 36.751 | 39.860 | 48.370 | 0.000 |
|  | TimeT3 | -2.768 | 0.877 | -4.489 | -1.048 | -3.157 | 0.002 |
|  | TimeT6 | -2.189 | 1.151 | -4.449 | 0.070 | -1.902 | 0.058 |
|  | TimeT12 | -1.434 | 1.195 | -3.779 | 0.912 | -1.200 | 0.231 |
|  | Age_groupOver 50 | -2.369 | 0.713 | -3.771 | -0.968 | -3.323 | 0.001 |
|  | Treatment_groupGalcanezumab | -0.992 | 0.913 | -2.786 | 0.803 | -1.086 | 0.278 |
|  | Treatment_groupFremanezumab | 1.018 | 0.999 | -0.945 | 2.981 | 1.019 | 0.309 |
|  | Treatment_groupEptinezumab | 1.271 | 1.214 | -1.115 | 3.657 | 1.047 | 0.296 |
|  | TimeT3:Age_groupOver 50 | 0.419 | 0.722 | -0.998 | 1.836 | 0.580 | 0.562 |
|  | TimeT6:Age_groupOver 50 | 0.919 | 0.918 | -0.883 | 2.720 | 1.001 | 0.317 |
|  | TimeT12:Age_groupOver 50 | 0.210 | 0.976 | -1.705 | 2.125 | 0.215 | 0.830 |
|  | TimeT3:Treatment_groupGalcanezumab | -0.225 | 1.004 | -2.196 | 1.745 | -0.224 | 0.823 |
|  | TimeT6:Treatment_groupGalcanezumab | -0.715 | 1.315 | -3.296 | 1.866 | -0.543 | 0.587 |
|  | TimeT12:Treatment_groupGalcanezumab | 1.532 | 1.353 | -1.123 | 4.187 | 1.132 | 0.258 |
|  | TimeT3:Treatment_groupFremanezumab | -0.152 | 1.076 | -2.264 | 1.960 | -0.141 | 0.888 |
|  | TimeT6:Treatment_groupFremanezumab | 1.273 | 1.384 | -1.444 | 3.990 | 0.919 | 0.358 |
|  | TimeT12:Treatment_groupFremanezumab | -1.009 | 1.443 | -3.841 | 1.824 | -0.699 | 0.485 |
|  | TimeT3:Treatment_groupEptinezumab | 1.503 | 1.246 | -0.942 | 3.948 | 1.207 | 0.228 |
|  | TimeT6:Treatment_groupEptinezumab | -1.089 | 1.556 | -4.143 | 1.966 | -0.700 | 0.484 |
|  | TimeT12:Treatment_groupEptinezumab | 0.051 | 1.728 | -3.342 | 3.443 | 0.029 | 0.977 |

Legend: BDI= Beck Depression Inventory; HIT-6=Headache Impact Test-6; MIDAS= Migraine Disability Assessment; MMD= Monthly Migraine Days; NRS= Numerical Rating Scale; SF36= Short Form 36

Supplementary Table 4: linear mixed model with only complete observations (sensitivity analysis)

| Outcome | Term | Estimate | Std.error | Conf.low | Conf.high | Statistic | p.value |
| --- | --- | --- | --- | --- | --- | --- | --- |
| MMD | (Intercept) | 18.308 | 1.029 | 16.287 | 20.329 | 17.790 | 0.000 |
|  | TimeT3 | -9.541 | 1.043 | -11.589 | -7.493 | -9.144 | 0.000 |
|  | TimeT6 | -8.192 | 1.043 | -10.240 | -6.144 | -7.851 | 0.000 |
|  | TimeT12 | -9.108 | 1.043 | -11.156 | -7.059 | -8.728 | 0.000 |
|  | Age_groupOver 50 | 1.164 | 0.862 | -0.528 | 2.856 | 1.351 | 0.177 |
|  | Treatment_groupGalcanezumab | -1.653 | 1.180 | -3.970 | 0.664 | -1.401 | 0.162 |
|  | Treatment_groupFremanezumab | -2.284 | 1.243 | -4.726 | 0.157 | -1.837 | 0.067 |
|  | Treatment_groupEptinezumab | 4.571 | 1.499 | 1.627 | 7.515 | 3.049 | 0.002 |
|  | TimeT3:Age_groupOver 50 | -1.770 | 0.874 | -3.485 | -0.056 | -2.027 | 0.043 |
|  | TimeT6:Age_groupOver 50 | -1.560 | 0.874 | -3.275 | 0.155 | -1.786 | 0.075 |
|  | TimeT12:Age_groupOver 50 | -0.476 | 0.874 | -2.190 | 1.239 | -0.544 | 0.586 |
|  | TimeT3:Treatment_groupGalcanezumab | 0.910 | 1.196 | -1.438 | 3.258 | 0.761 | 0.447 |
|  | TimeT6:Treatment_groupGalcanezumab | -1.303 | 1.196 | -3.651 | 1.045 | -1.089 | 0.276 |
|  | TimeT12:Treatment_groupGalcanezumab | -0.230 | 1.196 | -2.578 | 2.118 | -0.193 | 0.847 |
|  | TimeT3:Treatment_groupFremanezumab | 2.396 | 1.261 | -0.078 | 4.870 | 1.901 | 0.058 |
|  | TimeT6:Treatment_groupFremanezumab | -0.600 | 1.261 | -3.074 | 1.875 | -0.476 | 0.634 |
|  | TimeT12:Treatment_groupFremanezumab | -1.802 | 1.261 | -4.276 | 0.673 | -1.429 | 0.153 |
|  | TimeT3:Treatment_groupEptinezumab | -0.606 | 1.520 | -3.590 | 2.378 | -0.399 | 0.690 |
|  | TimeT6:Treatment_groupEptinezumab | -1.446 | 1.520 | -4.429 | 1.538 | -0.951 | 0.342 |
|  | TimeT12:Treatment_groupEptinezumab | -6.159 | 1.520 | -9.143 | -3.175 | -4.052 | 0.000 |
| Monthly acute medications | (Intercept) | 18.350 | 1.769 | 14.876 | 21.824 | 10.373 | 0.000 |
|  | TimeT3 | -10.811 | 1.819 | -14.382 | -7.241 | -5.944 | 0.000 |
|  | TimeT6 | -11.177 | 1.819 | -14.747 | -7.606 | -6.145 | 0.000 |
|  | TimeT12 | -10.915 | 1.819 | -14.486 | -7.345 | -6.002 | 0.000 |
|  | Age_groupOver 50 | 0.590 | 1.455 | -2.268 | 3.447 | 0.405 | 0.686 |
|  | Treatment_groupGalcanezumab | 0.742 | 2.014 | -3.212 | 4.696 | 0.369 | 0.713 |
|  | Treatment_groupFremanezumab | -2.770 | 2.128 | -6.949 | 1.409 | -1.301 | 0.194 |
|  | Treatment_groupEptinezumab | 6.195 | 2.535 | 1.217 | 11.173 | 2.444 | 0.015 |
|  | TimeT3:Age_groupOver 50 | -0.634 | 1.496 | -3.571 | 2.303 | -0.424 | 0.672 |
|  | TimeT6:Age_groupOver 50 | 0.920 | 1.496 | -2.017 | 3.857 | 0.615 | 0.539 |
|  | TimeT12:Age_groupOver 50 | -0.056 | 1.496 | -2.992 | 2.881 | -0.037 | 0.970 |
|  | TimeT3:Treatment_groupGalcanezumab | -1.443 | 2.070 | -5.506 | 2.621 | -0.697 | 0.486 |
|  | TimeT6:Treatment_groupGalcanezumab | -1.835 | 2.070 | -5.899 | 2.229 | -0.886 | 0.376 |
|  | TimeT12:Treatment_groupGalcanezumab | -1.956 | 2.070 | -6.020 | 2.108 | -0.945 | 0.345 |
|  | TimeT3:Treatment_groupFremanezumab | 2.065 | 2.188 | -2.231 | 6.360 | 0.944 | 0.346 |
|  | TimeT6:Treatment_groupFremanezumab | 0.914 | 2.188 | -3.381 | 5.210 | 0.418 | 0.676 |
|  | TimeT12:Treatment_groupFremanezumab | 0.008 | 2.188 | -4.288 | 4.303 | 0.003 | 0.997 |
|  | TimeT3:Treatment_groupEptinezumab | -1.202 | 2.606 | -6.318 | 3.915 | -0.461 | 0.645 |
|  | TimeT6:Treatment_groupEptinezumab | -1.007 | 2.606 | -6.123 | 4.109 | -0.386 | 0.699 |
|  | TimeT12:Treatment_groupEptinezumab | -0.197 | 2.606 | -5.313 | 4.919 | -0.076 | 0.940 |
| NRS | (Intercept) | 7.840 | 0.274 | 7.302 | 8.377 | 28.634 | 0.000 |
|  | TimeT3 | -1.750 | 0.339 | -2.415 | -1.084 | -5.162 | 0.000 |
|  | TimeT6 | -1.791 | 0.339 | -2.457 | -1.126 | -5.286 | 0.000 |
|  | TimeT12 | -1.670 | 0.339 | -2.335 | -1.004 | -4.926 | 0.000 |
|  | Age_groupOver 50 | 0.003 | 0.220 | -0.429 | 0.435 | 0.013 | 0.989 |
|  | Treatment_groupGalcanezumab | -0.117 | 0.311 | -0.727 | 0.493 | -0.376 | 0.707 |
|  | Treatment_groupFremanezumab | -0.162 | 0.325 | -0.799 | 0.475 | -0.500 | 0.617 |
|  | Treatment_groupEptinezumab | -0.003 | 0.385 | -0.759 | 0.753 | -0.008 | 0.994 |
|  | TimeT3:Age_groupOver 50 | -0.060 | 0.273 | -0.595 | 0.475 | -0.219 | 0.826 |
|  | TimeT6:Age_groupOver 50 | -0.069 | 0.273 | -0.604 | 0.466 | -0.254 | 0.800 |
|  | TimeT12:Age_groupOver 50 | 0.204 | 0.273 | -0.332 | 0.739 | 0.747 | 0.456 |
|  | TimeT3:Treatment_groupGalcanezumab | -0.185 | 0.385 | -0.940 | 0.570 | -0.481 | 0.630 |
|  | TimeT6:Treatment_groupGalcanezumab | -0.017 | 0.385 | -0.772 | 0.738 | -0.043 | 0.965 |
|  | TimeT12:Treatment_groupGalcanezumab | -0.486 | 0.385 | -1.241 | 0.269 | -1.263 | 0.207 |
|  | TimeT3:Treatment_groupFremanezumab | -0.133 | 0.402 | -0.922 | 0.656 | -0.331 | 0.741 |
|  | TimeT6:Treatment_groupFremanezumab | -0.284 | 0.402 | -1.073 | 0.505 | -0.706 | 0.480 |
|  | TimeT12:Treatment_groupFremanezumab | -0.350 | 0.402 | -1.138 | 0.439 | -0.870 | 0.385 |
|  | TimeT3:Treatment_groupEptinezumab | 0.287 | 0.477 | -0.648 | 1.223 | 0.603 | 0.547 |
|  | TimeT6:Treatment_groupEptinezumab | 0.387 | 0.477 | -0.549 | 1.323 | 0.812 | 0.417 |
|  | TimeT12:Treatment_groupEptinezumab | -0.089 | 0.477 | -1.024 | 0.847 | -0.186 | 0.853 |
| SF36 | (Intercept) | 107.445 | 4.286 | 99.030 | 115.859 | 25.071 | 0.000 |
|  | TimeT3 | -1.491 | 5.883 | -13.049 | 10.066 | -0.253 | 0.800 |
|  | TimeT6 | -15.276 | 5.883 | -26.834 | -3.719 | -2.597 | 0.010 |
|  | TimeT12 | -0.637 | 5.883 | -12.194 | 10.921 | -0.108 | 0.914 |
|  | Age_groupOver 50 | -3.001 | 2.864 | -8.625 | 2.623 | -1.048 | 0.295 |
|  | Treatment_groupGalcanezumab | -1.879 | 4.810 | -11.324 | 7.566 | -0.391 | 0.696 |
|  | Treatment_groupFremanezumab | -2.748 | 5.009 | -12.582 | 7.087 | -0.549 | 0.583 |
|  | Treatment_groupEptinezumab | -0.194 | 5.368 | -10.734 | 10.345 | -0.036 | 0.971 |
|  | TimeT3:Age_groupOver 50 | 4.614 | 3.932 | -3.111 | 12.338 | 1.173 | 0.241 |
|  | TimeT6:Age_groupOver 50 | -1.009 | 3.932 | -8.733 | 6.715 | -0.257 | 0.798 |
|  | TimeT12:Age_groupOver 50 | 4.821 | 3.932 | -2.904 | 12.545 | 1.226 | 0.221 |
|  | TimeT3:Treatment_groupGalcanezumab | -4.650 | 6.603 | -17.623 | 8.323 | -0.704 | 0.482 |
|  | TimeT6:Treatment_groupGalcanezumab | 14.511 | 6.603 | 1.539 | 27.484 | 2.198 | 0.028 |
|  | TimeT12:Treatment_groupGalcanezumab | -3.518 | 6.603 | -16.491 | 9.455 | -0.533 | 0.594 |
|  | TimeT3:Treatment_groupFremanezumab | -0.820 | 6.875 | -14.327 | 12.688 | -0.119 | 0.905 |
|  | TimeT6:Treatment_groupFremanezumab | 18.567 | 6.875 | 5.059 | 32.074 | 2.701 | 0.007 |
|  | TimeT12:Treatment_groupFremanezumab | -0.589 | 6.875 | -14.096 | 12.919 | -0.086 | 0.932 |
|  | TimeT3:Treatment_groupEptinezumab | -4.414 | 7.368 | -18.890 | 10.061 | -0.599 | 0.549 |
|  | TimeT6:Treatment_groupEptinezumab | 12.592 | 7.368 | -1.883 | 27.068 | 1.709 | 0.088 |
|  | TimeT12:Treatment_groupEptinezumab | -3.290 | 7.368 | -17.766 | 11.186 | -0.447 | 0.655 |
| MIDAS | (Intercept) | 84.165 | 7.611 | 69.218 | 99.113 | 11.058 | 0.000 |
|  | TimeT3 | -60.461 | 8.838 | -77.818 | -43.104 | -6.841 | 0.000 |
|  | TimeT6 | -64.206 | 8.838 | -81.563 | -46.850 | -7.265 | 0.000 |
|  | TimeT12 | -61.788 | 8.838 | -79.145 | -44.431 | -6.991 | 0.000 |
|  | Age_groupOver 50 | 2.608 | 5.294 | -7.789 | 13.005 | 0.493 | 0.622 |
|  | Treatment_groupGalcanezumab | 4.914 | 8.504 | -11.787 | 21.615 | 0.578 | 0.564 |
|  | Treatment_groupFremanezumab | -0.791 | 8.784 | -18.041 | 16.460 | -0.090 | 0.928 |
|  | Treatment_groupEptinezumab | 3.526 | 9.673 | -15.470 | 22.522 | 0.364 | 0.716 |
|  | TimeT3:Age_groupOver 50 | -8.310 | 6.147 | -20.382 | 3.762 | -1.352 | 0.177 |
|  | TimeT6:Age_groupOver 50 | -3.730 | 6.147 | -15.802 | 8.342 | -0.607 | 0.544 |
|  | TimeT12:Age_groupOver 50 | -0.787 | 6.147 | -12.860 | 11.285 | -0.128 | 0.898 |
|  | TimeT3:Treatment_groupGalcanezumab | -0.414 | 9.875 | -19.807 | 18.978 | -0.042 | 0.967 |
|  | TimeT6:Treatment_groupGalcanezumab | -4.779 | 9.875 | -24.172 | 14.614 | -0.484 | 0.629 |
|  | TimeT12:Treatment_groupGalcanezumab | -7.564 | 9.875 | -26.957 | 11.829 | -0.766 | 0.444 |
|  | TimeT3:Treatment_groupFremanezumab | 7.883 | 10.200 | -12.148 | 27.914 | 0.773 | 0.440 |
|  | TimeT6:Treatment_groupFremanezumab | 0.654 | 10.200 | -19.377 | 20.685 | 0.064 | 0.949 |
|  | TimeT12:Treatment_groupFremanezumab | -6.219 | 10.200 | -26.250 | 13.812 | -0.610 | 0.542 |
|  | TimeT3:Treatment_groupEptinezumab | 9.812 | 11.232 | -12.245 | 31.870 | 0.874 | 0.383 |
|  | TimeT6:Treatment_groupEptinezumab | 11.677 | 11.232 | -10.380 | 33.735 | 1.040 | 0.299 |
|  | TimeT12:Treatment_groupEptinezumab | 4.338 | 11.232 | -17.719 | 26.396 | 0.386 | 0.699 |
| HIT-6 | (Intercept) | 65.623 | 2.857 | 60.010 | 71.237 | 22.970 | 0.000 |
|  | TimeT3 | -11.433 | 3.282 | -17.881 | -4.984 | -3.484 | 0.001 |
|  | TimeT6 | -11.377 | 3.282 | -17.825 | -4.928 | -3.467 | 0.001 |
|  | TimeT12 | -7.750 | 3.282 | -14.198 | -1.301 | -2.361 | 0.019 |
|  | Age_groupOver 50 | -1.234 | 1.459 | -4.101 | 1.632 | -0.846 | 0.398 |
|  | Treatment_groupGalcanezumab | 0.040 | 3.072 | -5.997 | 6.076 | 0.013 | 0.990 |
|  | Treatment_groupFremanezumab | 0.082 | 3.164 | -6.135 | 6.299 | 0.026 | 0.979 |
|  | Treatment_groupEptinezumab | -1.174 | 3.348 | -7.751 | 5.404 | -0.351 | 0.726 |
|  | TimeT3:Age_groupOver 50 | -0.673 | 1.676 | -3.965 | 2.620 | -0.401 | 0.688 |
|  | TimeT6:Age_groupOver 50 | 0.768 | 1.676 | -2.524 | 4.061 | 0.458 | 0.647 |
|  | TimeT12:Age_groupOver 50 | 2.497 | 1.676 | -0.796 | 5.789 | 1.490 | 0.137 |
|  | TimeT3:Treatment_groupGalcanezumab | 2.919 | 3.529 | -4.016 | 9.853 | 0.827 | 0.409 |
|  | TimeT6:Treatment_groupGalcanezumab | 0.349 | 3.529 | -6.585 | 7.284 | 0.099 | 0.921 |
|  | TimeT12:Treatment_groupGalcanezumab | -5.784 | 3.529 | -12.719 | 1.150 | -1.639 | 0.102 |
|  | TimeT3:Treatment_groupFremanezumab | 3.187 | 3.634 | -3.954 | 10.329 | 0.877 | 0.381 |
|  | TimeT6:Treatment_groupFremanezumab | -0.574 | 3.634 | -7.715 | 6.568 | -0.158 | 0.875 |
|  | TimeT12:Treatment_groupFremanezumab | -3.894 | 3.634 | -11.035 | 3.247 | -1.071 | 0.285 |
|  | TimeT3:Treatment_groupEptinezumab | 6.458 | 3.845 | -1.098 | 14.015 | 1.679 | 0.094 |
|  | TimeT6:Treatment_groupEptinezumab | 5.274 | 3.845 | -2.282 | 12.830 | 1.371 | 0.171 |
|  | TimeT12:Treatment_groupEptinezumab | 1.906 | 3.845 | -5.650 | 9.462 | 0.496 | 0.620 |
| BDI | (Intercept) | 11.021 | 2.362 | 6.373 | 15.670 | 4.666 | 0.000 |
|  | TimeT3 | -4.996 | 1.987 | -8.901 | -1.092 | -2.514 | 0.012 |
|  | TimeT6 | -5.961 | 1.987 | -9.866 | -2.057 | -3.000 | 0.003 |
|  | TimeT12 | -2.911 | 1.987 | -6.816 | 0.993 | -1.465 | 0.144 |
|  | Age_groupOver 50 | -1.338 | 1.514 | -4.316 | 1.641 | -0.884 | 0.378 |
|  | Treatment_groupGalcanezumab | 2.746 | 2.649 | -2.468 | 7.959 | 1.036 | 0.301 |
|  | Treatment_groupFremanezumab | 2.815 | 2.785 | -2.666 | 8.295 | 1.011 | 0.313 |
|  | Treatment_groupEptinezumab | 5.577 | 2.928 | -0.185 | 11.338 | 1.905 | 0.058 |
|  | TimeT3:Age_groupOver 50 | -1.059 | 1.273 | -3.561 | 1.443 | -0.832 | 0.406 |
|  | TimeT6:Age_groupOver 50 | 0.383 | 1.273 | -2.119 | 2.884 | 0.301 | 0.764 |
|  | TimeT12:Age_groupOver 50 | 1.581 | 1.273 | -0.921 | 4.082 | 1.241 | 0.215 |
|  | TimeT3:Treatment_groupGalcanezumab | 2.214 | 2.229 | -2.165 | 6.593 | 0.993 | 0.321 |
|  | TimeT6:Treatment_groupGalcanezumab | 1.996 | 2.229 | -2.383 | 6.375 | 0.895 | 0.371 |
|  | TimeT12:Treatment_groupGalcanezumab | -1.462 | 2.229 | -5.841 | 2.917 | -0.656 | 0.512 |
|  | TimeT3:Treatment_groupFremanezumab | 3.655 | 2.343 | -0.948 | 8.258 | 1.560 | 0.119 |
|  | TimeT6:Treatment_groupFremanezumab | 3.975 | 2.343 | -0.628 | 8.578 | 1.696 | 0.090 |
|  | TimeT12:Treatment_groupFremanezumab | -1.010 | 2.343 | -5.613 | 3.593 | -0.431 | 0.667 |
|  | TimeT3:Treatment_groupEptinezumab | 2.476 | 2.463 | -2.363 | 7.315 | 1.005 | 0.315 |
|  | TimeT6:Treatment_groupEptinezumab | 4.041 | 2.463 | -0.798 | 8.881 | 1.641 | 0.101 |
|  | TimeT12:Treatment_groupEptinezumab | 1.119 | 2.463 | -3.720 | 5.959 | 0.454 | 0.650 |
| ZUNG | (Intercept) | 36.802 | 1.807 | 33.250 | 40.354 | 20.362 | 0.000 |
|  | TimeT3 | -3.073 | 1.920 | -6.844 | 0.698 | -1.601 | 0.110 |
|  | TimeT6 | -1.074 | 1.920 | -4.845 | 2.697 | -0.559 | 0.576 |
|  | TimeT12 | -1.251 | 1.920 | -5.022 | 2.520 | -0.652 | 0.515 |
|  | Age_groupOver 50 | -0.915 | 1.143 | -3.162 | 1.333 | -0.800 | 0.424 |
|  | Treatment_groupGalcanezumab | -0.198 | 2.011 | -4.151 | 3.755 | -0.098 | 0.922 |
|  | Treatment_groupFremanezumab | 1.417 | 2.106 | -2.721 | 5.556 | 0.673 | 0.501 |
|  | Treatment_groupEptinezumab | 2.132 | 2.222 | -2.235 | 6.499 | 0.959 | 0.338 |
|  | TimeT3:Age_groupOver 50 | -0.917 | 1.214 | -3.303 | 1.469 | -0.755 | 0.450 |
|  | TimeT6:Age_groupOver 50 | -0.411 | 1.214 | -2.797 | 1.975 | -0.339 | 0.735 |
|  | TimeT12:Age_groupOver 50 | 0.011 | 1.214 | -2.375 | 2.397 | 0.009 | 0.993 |
|  | TimeT3:Treatment_groupGalcanezumab | -0.548 | 2.136 | -4.744 | 3.649 | -0.256 | 0.798 |
|  | TimeT6:Treatment_groupGalcanezumab | -1.514 | 2.136 | -5.710 | 2.682 | -0.709 | 0.479 |
|  | TimeT12:Treatment_groupGalcanezumab | 0.783 | 2.136 | -3.414 | 4.979 | 0.366 | 0.714 |
|  | TimeT3:Treatment_groupFremanezumab | 0.954 | 2.236 | -3.440 | 5.348 | 0.427 | 0.670 |
|  | TimeT6:Treatment_groupFremanezumab | 1.005 | 2.236 | -3.389 | 5.399 | 0.449 | 0.653 |
|  | TimeT12:Treatment_groupFremanezumab | -0.969 | 2.236 | -5.363 | 3.425 | -0.433 | 0.665 |
|  | TimeT3:Treatment_groupEptinezumab | 0.674 | 2.360 | -3.962 | 5.310 | 0.286 | 0.775 |
|  | TimeT6:Treatment_groupEptinezumab | -1.994 | 2.360 | -6.631 | 2.642 | -0.845 | 0.398 |
|  | TimeT12:Treatment_groupEptinezumab | -0.571 | 2.360 | -5.208 | 4.065 | -0.242 | 0.809 |

Legend: BDI= Beck Depression Inventory; HIT-6=Headache Impact Test-6; MIDAS= Migraine Disability Assessment; MMD= Monthly Migraine Days; NRS= Numerical Rating Scale; SF36= Short Form 36

Supplementary table 5: generalized linear mixed-effects model results

|  | Estimate | Std.error | Statistic | p.value | Conf.low | Conf.high |
| --- | --- | --- | --- | --- | --- | --- |
| (Intercept) | -0.204 | 0.417 | -0.488 | 0.626 | -1.022 | 0.615 |
| Treatment_groupGalcanezumab | 1.943 | 0.605 | 3.211 | 0.001 | 0.757 | 3.129 |
| Treatment_groupFremanezumab | -0.387 | 0.425 | -0.911 | 0.362 | -1.219 | 0.446 |
| Treatment_groupEptinezumab | 1.249 | 0.764 | 1.635 | 0.102 | -0.249 | 2.747 |
| TimeT6 | -0.115 | 0.388 | -0.295 | 0.768 | -0.876 | 0.647 |
| TimeT12 | -0.061 | 0.421 | -0.145 | 0.884 | -0.885 | 0.763 |
| Age_groupOver 50 | 0.138 | 0.232 | 0.595 | 0.552 | -0.317 | 0.593 |
| Dose | -0.012 | 0.004 | -3.306 | 0.001 | -0.020 | -0.005 |
| Treatment_groupGalcanezumab:TimeT6 | 0.139 | 0.492 | 0.283 | 0.777 | -0.825 | 1.103 |
| Treatment_groupFremanezumab:TimeT6 | 0.665 | 0.528 | 1.261 | 0.207 | -0.369 | 1.699 |
| Treatment_groupEptinezumab:TimeT6 | 2.257 | 0.800 | 2.820 | 0.005 | 0.688 | 3.825 |
| Treatment_groupGalcanezumab:TimeT12 | 0.132 | 0.525 | 0.252 | 0.801 | -0.897 | 1.162 |
| Treatment_groupFremanezumab:TimeT12 | 1.775 | 0.588 | 3.019 | 0.003 | 0.623 | 2.927 |
| Treatment_groupEptinezumab:TimeT12 | 3.364 | 0.925 | 3.638 | 0.000 | 1.552 | 5.177 |

Supplementary Table 6: prediction of treatment response with age as a continuous variable (sensitivity analysis)

|  | Estimate | Std.error | Statistic | p.value | Conf.low | Conf.high |
| --- | --- | --- | --- | --- | --- | --- |
| (Intercept) | -0.642 | 0.618 | -1.040 | 0.298 | -1.853 | 0.568 |
| Treatment_groupGalcanezumab | 1.960 | 0.605 | 3.237 | 0.001 | 0.773 | 3.146 |
| Treatment_groupFremanezumab | -0.410 | 0.425 | -0.964 | 0.335 | -1.244 | 0.424 |
| Treatment_groupEptinezumab | 1.277 | 0.765 | 1.669 | 0.095 | -0.222 | 2.777 |
| TimeT6 | -0.114 | 0.389 | -0.294 | 0.768 | -0.876 | 0.647 |
| TimeT12 | -0.060 | 0.421 | -0.141 | 0.888 | -0.884 | 0.765 |
| Age | 0.011 | 0.010 | 1.068 | 0.286 | -0.009 | 0.030 |
| Dose | -0.013 | 0.004 | -3.331 | 0.001 | -0.020 | -0.005 |
| Treatment_groupGalcanezumab:TimeT6 | 0.142 | 0.492 | 0.288 | 0.773 | -0.823 | 1.106 |
| Treatment_groupFremanezumab:TimeT6 | 0.663 | 0.528 | 1.256 | 0.209 | -0.371 | 1.697 |
| Treatment_groupEptinezumab:TimeT6 | 2.266 | 0.800 | 2.832 | 0.005 | 0.697 | 3.834 |
| Treatment_groupGalcanezumab:TimeT12 | 0.131 | 0.525 | 0.249 | 0.803 | -0.899 | 1.161 |
| Treatment_groupFremanezumab:TimeT12 | 1.769 | 0.588 | 3.010 | 0.003 | 0.617 | 2.921 |
| Treatment_groupEptinezumab:TimeT12 | 3.361 | 0.924 | 3.638 | 0.000 | 1.550 | 5.172 |

Supplementary Table 7: prediction of treatment response with additional covariates (sensitivity analysis)

|  | estimate | std.error | statistic | p.value | conf.low | conf.high |
| --- | --- | --- | --- | --- | --- | --- |
| (Intercept) | 0.379 | 0.620 | 0.611 | 0.541 | -0.836 | 1.593 |
| Treatment_groupGalcanezumab | 1.762 | 0.617 | 2.857 | 0.004 | 0.553 | 2.972 |
| Treatment_groupFremanezumab | -0.566 | 0.449 | -1.260 | 0.208 | -1.445 | 0.314 |
| Treatment_groupEptinezumab | 1.199 | 0.772 | 1.553 | 0.120 | -0.314 | 2.713 |
| TimeT6 | -0.125 | 0.410 | -0.304 | 0.761 | -0.928 | 0.679 |
| TimeT12 | 0.045 | 0.445 | 0.100 | 0.920 | -0.827 | 0.916 |
| Age_groupOver 50 | 0.241 | 0.268 | 0.899 | 0.369 | -0.284 | 0.765 |
| T0_MMD | -0.011 | 0.016 | -0.691 | 0.490 | -0.043 | 0.020 |
| Number of previous prophilaxis treatments | -0.089 | 0.069 | -1.292 | 0.196 | -0.225 | 0.046 |
| History of migraine (years) | -0.005 | 0.010 | -0.467 | 0.640 | -0.025 | 0.016 |
| DiagnosisCM | 0.279 | 0.244 | 1.141 | 0.254 | -0.200 | 0.757 |
| Dose | -0.012 | 0.004 | -3.309 | 0.001 | -0.020 | -0.005 |
| Treatment_groupGalcanezumab:TimeT6 | 0.154 | 0.508 | 0.304 | 0.761 | -0.841 | 1.150 |
| Treatment_groupFremanezumab:TimeT6 | 0.662 | 0.542 | 1.221 | 0.222 | -0.400 | 1.725 |
| Treatment_groupEptinezumab:TimeT6 | 2.231 | 0.805 | 2.773 | 0.006 | 0.654 | 3.808 |
| Treatment_groupGalcanezumab:TimeT12 | 0.025 | 0.543 | 0.046 | 0.963 | -1.040 | 1.090 |
| Treatment_groupFremanezumab:TimeT12 | 1.629 | 0.602 | 2.705 | 0.007 | 0.449 | 2.810 |
| Treatment_groupEptinezumab:TimeT12 | 3.200 | 0.927 | 3.451 | 0.001 | 1.382 | 5.017 |

Supplementary table 8: simplified generalized linear mixed-effects model results for the age ≥65 subgroup

| Term | Estimate | Std.error | Statistic | p.value | Conf.low | Conf.high |
| --- | --- | --- | --- | --- | --- | --- |
| (Intercept) | 0.713 | 0.153 | 4.662 | 0.000 | 0.413 | 1.013 |
| TimeT6 | 0.224 | 0.184 | 1.217 | 0.224 | -0.137 | 0.585 |
| TimeT12 | 0.685 | 0.203 | 3.376 | 0.001 | 0.287 | 1.083 |
| Over65 | -1.150 | 0.793 | -1.451 | 0.147 | -2.704 | 0.403 |
| TimeT6:Over65 | 0.338 | 0.964 | 0.350 | 0.726 | -1.552 | 2.227 |
| TimeT12:Over65 | -0.469 | 0.987 | -0.476 | 0.634 | -2.403 | 1.464 |

Supplementary table 9: factor loadings from Principal Component Analysis with oblimin rotation

| **Variable** | **Component 1: General Improvement** | **Component 2: Quality of life vs mood** |
| --- | --- | --- |
| ΔMIDAS T3 | 0.707 | 0.188 |
| ΔBDI T3 | 0.665 | -0.302 |
| ΔHIT-6 T3 | 0.633 | 0.193 |
| ΔZung T3 | 0.584 | -0.097 |
| ΔSF36 T3 | 0.195 | 0.576 |
| ΔMIDAS T6 | 0.730 | 0.153 |
| ΔBDI T6 | 0.620 | -0.322 |
| ΔHIT-6 T6 | 0.512 | 0.117 |
| ΔZung T6 | 0.445 | -0.074 |
| ΔSF36 T6 | -0.030 | 0.655 |
| ΔMIDAS T12 | 0.731 | 0.141 |
| ΔBDI T12 | 0.650 | -0.243 |
| ΔHIT-6 T12 | 0.593 | 0.172 |
| ΔZung T12 | 0.410 | -0.285 |
| ΔSF36 T12 | 0.072 | 0.653 |

Legend: BDI= Beck Depression Inventory; HIT-6=Headache Impact Test-6; MIDAS= Migraine Disability Assessment; MMD= Monthly Migraine Days; NRS= Numerical Rating Scale; SF36= Short Form 36

Supplementary figure 1: Principal Component Analysis Scree Plot

Supplementary figure 2: Principal Component 1 *General improvement* differences across treatment types

PC1=Principal Component 1

* p<0.05 (*Post hoc* Dunn tests with Bonferroni correction)

Supplementary figure 3: Principal Component 2 *Quality of life versus mood* differences across treatment types

PC2=Principal Component 2

* p<0.05 (*Post hoc* Dunn tests with Bonferroni correction)
